# Assessing the competitiveness of solar photovoltaic products in comprehensive and progressive agreement for trans-pacific partnership countries

**DOI:** 10.1371/journal.pone.0284783

**Published:** 2023-07-27

**Authors:** Qing Guo, Wenlan You

**Affiliations:** School of Economics and Trade, Guangdong University of Foreign Studies, Guangzhou, Guangdong Province, China; Taylor’s University - Lakeside Campus: Taylor’s University, MALAYSIA

## Abstract

Solar PV enables the conversion of solar energy into electricity and has become a clean energy technology for economic development. The advantages and disadvantages of solar photovoltaic vary among CPTPP member nations; nevertheless, since the CPTPP’s implementation, fewer researchers have evaluated the member countries’ solar photovoltaic goods’ level of international competitiveness. In order to correct the flaw that the RCA index can only be studied from trade volume, this study adopts the IRCA index method and the revised CMS model, which integrates a number of variables to analyze the competitiveness of solar photovoltaic in each country. The study’s findings indicate that: (1) the structural effect has a significant impact on export growth, particularly during the epidemic period, when Australia’s contribution rate reached 106.58%; (2) the competitiveness effect has a lesser impact on export growth, and the epidemic led to a decline in competitiveness, with Japan’s contribution rate reaching -262.85%; (3) the second-order effect has a declining trend in its contribution rate of export growth; (4) the export competitiveness of solar PV products differs between CPTPP countries, with power supply products having the most export competitiveness and solar cells having a steady comparative advantage in a weaker state. Apart than Japan, the remaining nations’ comparative advantages for PV products are less than 1. Based on the above research findings, this paper puts forward the corresponding policy recommendations.

## 1. Introduction

The two historical industrial revolutions have consumed large amounts of fossil energy and also emitted large amounts of greenhouse gases. Rapid development of renewable energy industry helps alleviate energy poverty [1]. The depletion of resources and environmental pollution caused by excessive consumption of fossil energy has made the development and utilization of renewable energy the focus of the world [2]. Renewable energy provides the answer to the most pressing socio-economic challenges facing governments today, especially the impact of climate change [3]. Solar photovoltaic (PV) power is a new and green energy source, global concerns about environment and climate change have led to the rapid development of solar PV industry across the world. The Comprehensive and Progressive Trans-Pacific Partnership (CPTPP), a free trade area for Asia-Pacific countries [4], is the new name for the agreement following the withdrawal of the United States from the Trans-Pacific Partnership (TPP). Five chapters of the CPTPP provisions deal with trade-related environmental issues, with key words related to environmental protection and sustainable development appearing over 140 times, It is clear that the CPTPP countries value trade in renewable energy sources, and it is anticipated that trade in renewable energy-related goods would grow in the future. However, the CPTPP countries have different strengths and problems in renewable energy. For example, Vietnam has a good renewable energy endowment and huge development potential, but it is still in its infancy due to technical limitations, financing constraints and weak policies [5]; Singapore struggles with a lack of renewable energy, so finding ways to meet its energy needs, advance sustainable development, and combat climate change has been a priority [6]. Japan’s mature new energy development rate and high module market prices are favoured by overseas PV companies, but the flat land area suitable for solar PV is limited and a large number of power plants are located in disaster risk areas; Although New Zealand’s proportional contribution to the entire energy mix is relatively minor and it still faces energy security and mitigation challenges, it has the third highest share of renewable energy in total primary energy supply among OECD countries; Peru is a diverse nation with the potential to develop renewable energy sources and the need to preserve its natural heritage. Yet, there is not enough room for urban renewable energy development, which slows the industry’s rapid expansion; Mexico has abundant solar energy resources, and there are numerous large desert areas that are suited for solar power. Nevertheless, the current Mexican government announced the cancellation of the fourth energy auction, which casts doubt on the PV industry’s future growth. The CPTPP countries are vast and strategically located, with abundant non-fossil energy sources such as wind, solar, hydro, biomass and geothermal energy. The trade agreement is generally considered an effective mechanism to encourage trading activities [7], green governance is increasingly becoming a standard feature of today’s regional trade agreements [8]. With its unlimited supply, low operating costs, safety and dependability, lack of negative environmental effects, and other inherent benefits over other renewable energy sources, solar photovoltaic has emerged as the renewable energy source of choice for many CPTPP nations. It is expected that in the future, solar photovoltaic products will play a significant role in the exports of CPTPP nations.

**Fig 1:**
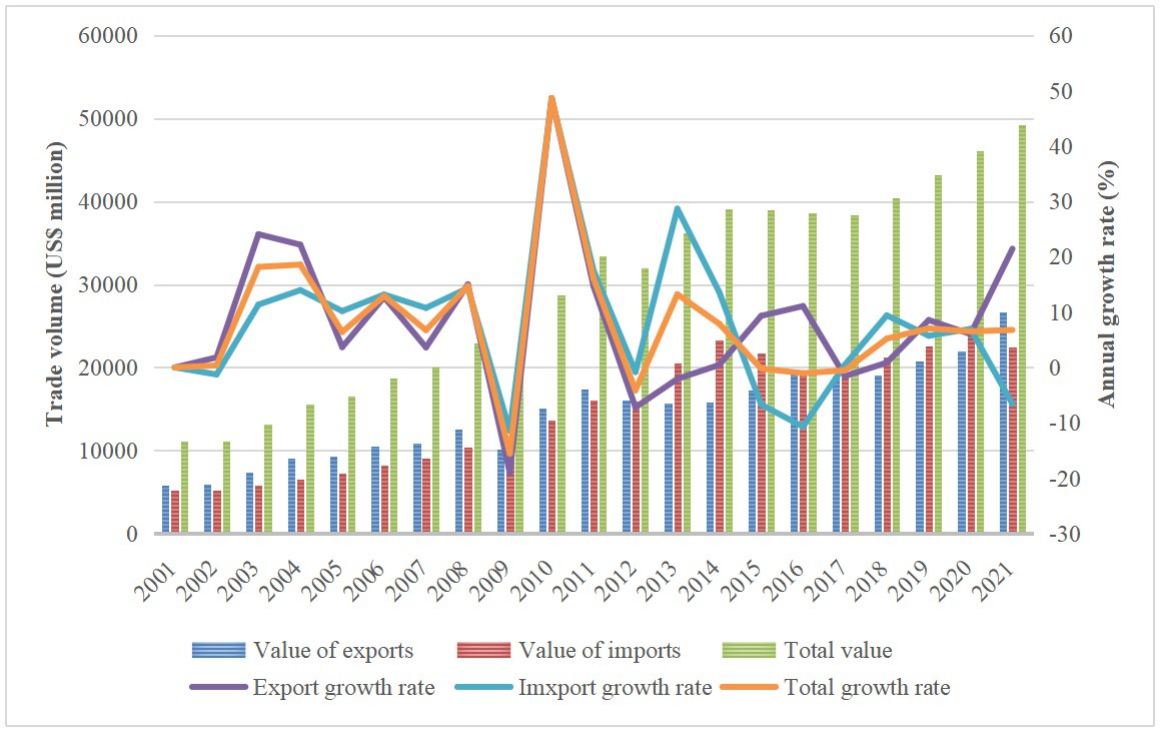
Changes in the structure of solar PV product exports from CPTPP countries (Data obtained from UN Comtrade).

**Fig 2:**
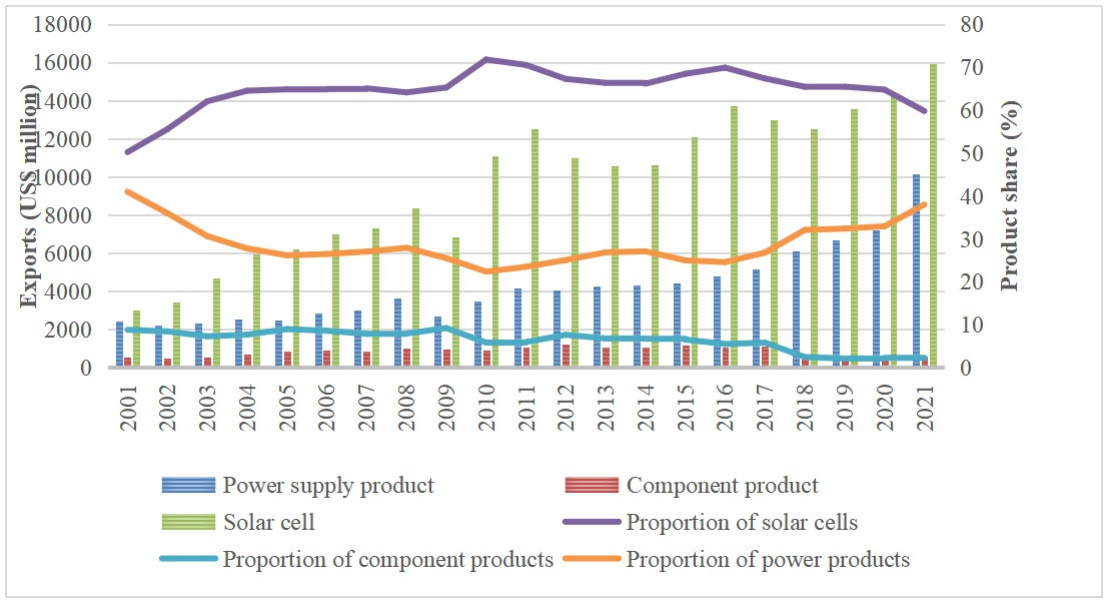
Changes in the export structure of solar PV products from CPTPP countries (Data obtained from UN Comtrade).

According to the United Nations trade database (UN comtrade) database, the trade in solar PV products shows an overall rising trend for CPTPP countries, with exports rising from US$10,931.87 million in 2007 to US$2,6699.25 million in 2021, with an annual growth rate of 144.23%; and imports rising from US$9072 million to US$22510.83 million in 2016, with an annual growth rate of 148.13%(Data on solar PV products in Brunei and New Zealand are not available due to their low availability) (Fig 1). In terms of the export structure of solar PV products from CPTPP countries, the share of solar cell exports was much higher than that of the other two products during 2001–2017, while the share of module products has been the lowest. 2016 solar cell exports accounted for a higher share of solar PV product exports, up to 69.91%. In contrast, the export value of module products accounted for about 7%. The export value of power supply products accounted for 26% (Fig 2). According to the dynamic changes, the export value of module products and power supply products, which make up a percentage of the exports of solar photovoltaic products, showed a tendency of dropping and then rising over the period of 2001–2017. The fraction of 2021 is lower than that of 2001, the proportion of module goods increased and then decreased, and the overall proportion of solar cell exports increased from 49% to 59.79%.

Solar photovoltaic power generation is of great importance to CPTPP countries. We shall address the competitiveness and comparative advantages of PV products in the CPTPP countries in this essay, as well as the effects of shifting PV product market and export structures on the development of each nation’s exports. The main methods used to gauge a nation’s product competitiveness are the TC index and RCA index. However, all of these metrics share a common flaw: they only take into account a nation’s import and export data, omitting the impact of economic development or scientific and technological advancement, both of which have a greater impact on a nation’s product export competitiveness. As a result, based on the conventional RCA index, we combine the CMS model and the IRCA index to create an assessment index system that allows us to thoroughly examine the factors that contribute to the export volatility of solar PV products in CPTPP nations. The research advances knowledge in the fields of solar photovoltaics and global competitiveness and offers CPTPP member nations a theoretical framework for developing PV policies.

## 2. Review of the literature

A search of the relevant literature reveals that more research has been carried out in academia on trade in renewable energy products and less on solar PV products. Specifically, in terms of research content, it can be divided into four main areas.

### 2.1. Current status of trade in renewable energy products

Vo et al. [9] used second generation estimation analysis to examine the joint and independent effects of financial development and renewable energy on energy consumption in CPTPP countries over the period 1971–2019, and the results confirmed the existence of an inverted ’U’ shaped relationship between energy consumption and economic growth. Zhu et al. [10] constructed a global trade network of key minerals from 2000–2019 and used a dynamic econometric model to analyse the impact of trade patterns on renewable energy development. The results showed that trade strength and central influence can contribute to renewable energy development through technological advances in renewable energy. Fu and Wu [11] applied an extended gravity model to analyse the impact of environmental regulations and the institutional quality of importing and exporting countries and Chinese renewable energy R&D expenditure on the depth and breadth of exports in the Chinese solar and wind energy sectors. The results showed that renewable energy R&D expenditure only has a significant effect on the depth of exports in the solar industry. Chowdhury et al. [12] reviewed the current status of solar panel waste recycling, recycling technologies, environmental protection, waste management, recycling policies and economic aspects of recycling, arguing that the need to further improve the economic viability, practicality, high recycling rates and environmental performance of the PV industry is essential. Shuai et al. [13] constructed a new multidimensional evaluation indicator to estimate the international competitiveness of renewable energy products exported by China, the US and Indiac. Al-Shahri et al. [14] showed that the intermittent nature of electricity generation may affect the reliability of electricity and the potential for thermal power plants to in turn affect the ecosystem if they are not managed properly, among other issues. Hossain et al. [15] stated that Malaysia has a potential of 29,000 MW to develop hydropower, which is currently only 11% developed, mainly due to the high cost of equipment, the general resistance of the population to building dammed hydropower plants, and the lack of technical support to build hydropower plants. Lau et al. [16] stated that ASEAN solar energy has better sustainability compared to bioenergy and hydro energy, but the contribution of renewables to ASEAN has been declining. Daren [17] argued that renewables provide 18% of total electricity generation in the US and that this impressive growth is driven by robust financial markets.

### 2.2. Policy recommendations for the renewable energy industry

Gielen et al. [18] found that energy efficiency and renewable energy technologies are core elements to accelerate the energy transition and that economic conditions, resources, science and technology and socioeconomic effects can effectively support the world energy transition. Calise et al. [19] suggested that the government should implement new energy funding subsidies or tax breaks to support the development of various sectors, with a focus on incentives. Wang et al. [20] suggested that China should accelerate the research and development of renewable energy technologies through fiscal investment, providing loan subsidies and investment incentives to encourage renewable energy production, government purchase and consumption subsidy policies. Boke et al. [21] recommended that Ethiopia’s energy mix continue to be dominated by hydropower and begin a gradual shift towards solar and wind energy development as the lowest cost energy supply options. Mirletz et al. [22] stated that in order to reduce the material requirements and life-cycle waste incurred in achieving decarbonization goals, manufacturing waste recycling should be increased in an effort to recover valuable photovoltaic materials. Zhao et al. [23] pointed out that lowering tariffs not only encourages entry into new export markets in general, but also leads to an increase in the number of renewable energy products already traded. Moreover, the positive impact of trade liberalization is more pronounced for foreign exporters than for state-owned or private exporters.

### 2.3 Research on CPTPP

Chang [24] found that the GVC intensity of trade within the CPTPP group of countries is higher than their overall trade, and that GVC connectivity within the CPTPP group will further increase with the entry into force of the agreement. Qian [25] pointed out that the CPTPP is an important development in deepening trade and investment linkages in the Asia-Pacific region, and provides a new and unique regional trade features. Its normative quality and geographical scope make it one of the most detailed and important investment agreements. Jung [26] quantified the impact of China’s participation in the CPTPP. The results suggested that China’s participation in the CPTPP may generate higher productivity, GDP, and welfare effects than previous traditional CGE models based on a simplistic representative agent framework. Marcoux [27] argued that China is challenging the emerging norms regarding SOEs and general partners through the Belt and Road, which has serious implications for its possible accession to the CPTPP. Chaisse et al. [28] discussed many aspects of the paradigm shift brought about by the TPP, stating that it might start a revolution in international trade/economic law and potentially alter how trade and investment are done.

### 2.4. Research on trade in solar PV products

Sun [29] used Porter’s diamond model to analyse the PV industry in four countries, China, the US, Germany and Japan, and concluded that the PV industry in Europe and the US had developed earlier and had a more complete industrial system, and had a clear advantage in R&D and technology in the PV industry, and was very competitive in the international market. Wang et al. [30] evaluated the hindering effect of tariff barriers on global PV product trade and emission reduction based on a technology-based integrated dynamic predictive electricity market share model. Shuai et al. [31] evaluated the complementarity of solar PV trade between China and the United States using the trade combination index, export similarity index, and trade complementarity index. Zhao et al. [32] analyzed the pattern of international trade in solar PV cells from 1996–2015 in both spatial and temporal terms.

Scholars have already used the diamond model and TCI to study the competitiveness of solar PV products. However, there is less empirical literature on competitiveness studies for specific objects like CPTPP countries, and most studies only refer to the trade volume and do not include the factors that may affect the competitiveness of PV products into the evaluation system. Compared with the existing literature, the possible innovations of this paper are mainly: first, based on the conventional RCA index, the evaluation index system is intended to create the IRCA index in order to thoroughly investigate the causes of the variation in solar PV product exports of CPTPP countries. These are the main possible innovations of this paper in comparison to the existing literature. To measure the long-term trend of the international competitiveness of solar PV products in CPTPP countries, an improved CMS model is used. The goal is to identify the main drivers of export growth as well as the main factors that are limiting that growth in order to make practical recommendations for fostering the growth of solar PV exports in CPTPP countries.

## 3. Research methodology and data sources

### 3.1. Research methodology

#### 3.1.1 IRCA index

In this paper, a number of variables reflecting the competitiveness of PV products are downscaled by the projection tracing method to obtain weights, and multiplied with the traditional RCA index to construct a new IRCA index. Specifically, it is shown in Eq (1)

$$IRCA_{ij}=a_{i}RCA_{ij}$$ (1)

where *a* denotes the weight and *i*, *j* denote the *i-th* period in the *j-th* commodity group, respectively. In a mathematical sense, the weight *a* is a correction made to the export volume. The acquisition of the traditional RCA index and weights is described next.

(1) Traditional RCA Index

Balassa proposed the RCA (revealed comparative advantage index), which is commonly used to measure the comparative advantage of a country’s industry in international trade. In order to quantitatively analyse the comparative advantage of different products of solar photovoltaic products exported by CPTPP countries, this paper will use the RCA method to analyse the comparative advantage of three products, namely power supply products, module products and solar cells exported by CPTPP countries, based on relevant data retrieved from UN comtrade during 2001–2021.

The calculation of the RCA of industry *j* in country *i* in international trade refers to the formula in the article by scholars Li and Wu [33], as in Eq (2).


$$RCA=\frac{X_{ij}/X_{i}}{X_{wj}/X_{w}}$$ (2)

*Xij* denotes the export value of industry *j* in country *i*, *X*_*i*_ denotes the export value of goods in country *i*, *Xwj* denotes the export value of industry *j* worldwide, and *X*_*w*_ denotes the export value of goods worldwide. If RCA_*ij*_ <1, industry *j* in country *i* is at a comparative disadvantage in international trade; if RCA_*ij*_ >1, it is at a comparative advantage, and the larger the value, the greater the comparative advantage.

As the international trade environment changes and science and technology continue to innovate, some scholars have found that the traditional RCA index method has obvious limitations in scientometrics research [34]. The limitations of the RCA index method are that it focuses more on static analysis, so it cannot be used to predict the trend of a country’s trade development, and it also does not take into account the fact that a country’s It also fails to take into account the fact that a country’s resource endowment or even comparative advantage is in fact evolving, and to some extent, the important role of import trade in a country’s foreign trade and industrial development [35]. This paper attempts to overcome the weaknesses of the traditional RCA index by using relevant indicators and constructing weights to be added to the traditional RCA index.

(2) Weights

The projection tracing method is used in this paper to generate weights. A group of statistical techniques known as projection tracing are used to analyse and analyze high-dimensional data. In order to analyze high-dimensional data, the main idea is to project high-dimensional data onto a low-dimensional subspace and discover a projection that accurately captures the structure or properties of the original high-dimensional data [36]. It has the advantages of strong objectivity and robustness [37]. This study uses this method to transform multiple variables related to export competitiveness, i.e. p-dimensional data {x(i,j)|j = 1,2,…, p} into one-dimensional data, i.e. innovation weights a = {a(1),a(2),a(3),…, a(p)}, where the larger the coefficient of a variable prediction vector, the greater the influence of that variable.

① Selection of indicators

Based on the availability of data, this paper identifies five specific factors that affect the trade flows of solar PV products in CPTPP countries, namely: total population, GDP, solar power generation (GWh), renewable energy consumption as a percentage of end-use energy consumption, and total renewable energy generation (GWh) in each country from 2001–2017.

② Projection tracing process

A. Normalization of the sample evaluation indicator set

In this study, the data were normalized using the min-max normalization method with the following transformation function for the series *x*. Let the value of the jth evaluation index of the ith transect be *x*_*ij*_, min(*x*_*j*_), max(*x*_*j*_) are the minimum and maximum values in the sample data of the jth evaluation index. The specific conversion formula is shown in Eq (3). Eqs 3–11 refers to the article by Zhang and other scholars [38].


$$y_{ij}=\frac{\left(x_{ij}-min\left(x_{j}\right)\right)}{\left(max\left(x_{j}\right)-min\left(x_{j}\right)\right)}$$ (3)

B. Construction of the projection objective function

Multiply the p-dimensional data x(i,j) synthetically by a = {a(1),a(2),a(3),…, a(p)}, the obtained one-dimensional projection value *Z*_*i*_ is as in Eq (4):

$$Z_{i}=\overset{p}{\underset{j=1}{\sum }}a_{j}x_{i}{}_{j}$$ (4)

where *a* is the unit length vector. The projection needs to be analysed as a scatter diagram, which requires the local cohesion to be shown as far as possible while ensuring that the cohesion points are spread out from each other, So the projection indicator function *Q*(*a*) is as in Eq (5):

$$Q\left(a\right)=S\left(a\right)D\left(a\right)$$ (5)

$$S\left(a\right)=\sqrt{\frac{1}{n}\overset{n}{\underset{j=1}{\sum }}{\left(z_{i}-\bar{z}\right)}^{2}}$$ (6)

$$D\left(a\right)=\sum_{i=1}^{n}\sum_{j=1}^{p}\left(R-r_{ij}\right)f\left(R-r_{ij}\right)$$ (7)

Where *S*(*a*) in Eq (6) is the standard deviation of the local density, *D*(*a*) in Formula 6 represents local density. *z* is the sequence, *R* is the window radius of the local density, and in this study, *r*_*ij*_ in Eq (8) is the inter-sample distance, and *f*(*x*) in Eq (9) is the step signal.


$$r_{ij}=\left|z_{i}-\right.\left.z_{j}\right|$$ (8)

$$f\left(x\right)=\left\{\begin{array}{ll} 0, & x<0\\ 1, & x\geq 0 \end{array}\right.$$ (9)

C. Optimisation of the projection indicator function

After the sample data are determined, the projection direction vector a is the single variable that affects the projection indicator function Q(a), so the optimal projection vector finding problem can be equated to solving for the maximum value of the projection indicator function.

Objective function is as in Eq (10):

$$\text{max}\left(Q\left(a\right)\right)=S\left(a\right)D\left(a\right)$$ (10)

Binding conditions is Formula (11):

$$s.t.\;\;\overset{p}{\underset{j=1}{\sum }}a_{j}^{2}=1$$ (11)

The derived *a* = {a(1),a(2),a(3),…, a(p)} are the weights for this study.

#### 3.1.2. Constant market share (CMS)

(1) Introduction to the model

The CMS model was proposed by Tyszynski in 1951 and later improved by several scholars such as Jepma (1989) to become a practical and more accurate tool for analyzing the factors influencing trade volatility, which is widely used in international trade analysis. Liu and Li [39] argued that CMS model is one of the commonly used methods to study the change of world trade structure and the main reasons for the growth of export trade of a single country. Qin et al. [40] believed that CMS model is an important tool to analyze the dynamic changes of export and its influencing factors. Zhang and Zhang [41] studied the causes of trade fluctuations in Chinese sunflowers based on the CMS model, Zhe and Han [42] used the CMS model to study the factors of trade fluctuations in Chinese ceramic exports, Ge et al. [43] constructed the CMS three-level decomposition model to study the drivers of China’s agricultural export growth under the RCEP framework. Domestic and foreign studies also showed that this model has a strong ability to explain the decomposition of export growth. Therefore, this paper adopts CMS model to study the competitiveness of CPTPP countries’ export solar photovoltaic products.

**Fig 3:**
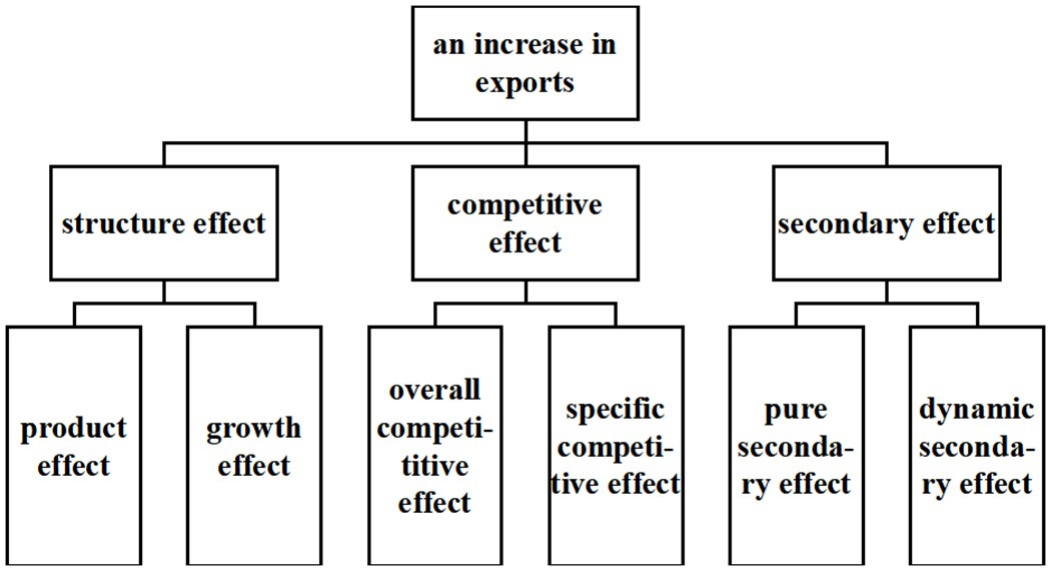
Schematic of the CMS model’s two-level decomposition of export growth.

The model makes the assumption that if a country’s solar PV export competitiveness stays consistent, its market share should also theoretically be the same. Hence, changes in competitiveness or export structure must be the reason for the discrepancy between real changes in a country’s exports and changes in its competitors’ exports. Based on this supposition and statistical principles, the CMS model divides export growth into two categories and compares the value, commodity structure, and market structure (sample) of exports from CPTPP nations with the rest of the world during the same time period. Second-level effects are further broken down into growth effects, product effects, general competitiveness effects, specific competitiveness effects, pure second-order effects, and dynamic second-order effects at the second level. The first level is divided into structural, competitive, and second-order effects. By analysing the share of the different effects in the growth of exports of goods, we can easily find the share of the contribution of the CPTPP countries to the competitiveness of solar PV exports and reveal the sources of export growth and the factors that constrain growth. The framework of this model is shown in Fig 3.

(2) Decomposition formula

① One layer decomposition formula is Eq (12):

$$\Delta q=\underset{i}{\sum }s_{i}^{0}\Delta Q_{i}+\underset{i}{\sum }s_{i}Q_{i}^{0}\;+\underset{i}{\sum }\Delta s_{i}\Delta Q_{i}$$ (12)

The first level of this modified CMS model splits the total effect of export growth into structural, competitiveness and second-order effects. $\sum_{i}s_{i}^{0}\Delta Q_{i}$ is the structural effect, representing the change in the export value of solar PV products of a CPTPP country due to a change in the total imports of solar PV products in the world market. $\sum_{i}{\Delta s}_{i}Q_{i}^{0}$ is the competitiveness effect, indicating the change in the export value of solar PV products of a CPTPP country due to a change in the competitiveness of solar PV product exports of that country. ∑_*i*_Δ*s*_*i*_Δ*Q*_*i*_ is a second-order effect, representing the change in the value of solar PV exports of a country in the CPTPP due to a change in both total solar PV imports in the world market and the competitiveness of solar PV exports of a country in the CPTPP.

② Two-level decomposition formula is as Eq (13):

$$\begin{array}{ll} \Delta q & =s^{0}\Delta Q+\left(\sum_{i}s_{i}^{0}\Delta Q_{i}-s^{0}\Delta Q\right)+\Delta sQ^{0}+\left(\sum_{i}s_{i}Q_{i}^{0}-\Delta sQ^{0}\right)\\ & +\left[\left(Q^{1}/Q^{0}-1\right)\sum_{i}s_{i}Q_{i}^{0}\right]+\left[\sum_{i}\Delta s_{i}\Delta Q_{i}-\left(Q^{1}/Q^{0}-1\right)\sum_{i}s_{i}Q_{i}^{0}\right] \end{array}$$ (13)

In the above equation, *q* denotes the export value of solar PV products to the world from a CPTPP country; *Q* denotes the total import value of solar PV products to the world. *Q*_*i*_ denotes the total amount of world imports of solar photovoltaic products of type *i*; *s* denotes the share of the export amount of solar photovoltaic products of a CPTPP country to the world in the total amount of world imports of solar photovoltaic products. *s*_*i*_ denotes the share of the export value of solar PV products of category *i* of a CPTPP country in the total world import value of solar PV products of that category. *s*^0^Δ*Q* denotes the growth effect. $\left(\sum_{i}s_{i}^{0}\Delta Q_{i}-s^{0}\Delta Q\right)$ denotes the product effect. Δ*sQ*^0^ denotes the overall competitive effect. $\left(\sum_{i}s_{i}Q_{i}^{0}-\Delta sQ^{0}\right)$ denotes the specific competition effect. $\left[\left(Q^{1}/Q^{0}-1\right)*\sum_{i}s_{i}Q_{i}^{0}\right]$ denotes the pure second-order effect. $\left[\sum_{i}{\Delta s}_{i}\Delta Q_{i}-\left(Q^{1}/Q^{0}-1\right)*\sum_{i}s_{i}Q_{i}^{0}\right]$ denotes the dynamic second-order effects; 0 and 1 represent the base period and end period respectively; Δ denotes the amount of change in both periods. Eqs 11 and 12 refer to the article of scholars Liu and Li [44].

### 3.2 Data sources

Solar photovoltaic products mainly include solar cells, power supply products and module products, whose HS codes are 854140, 850440 and 841990 respectively. According to the classification method of the International Convention on Harmonized System, the above solar photovoltaic products trade data of CPTPP countries and the world from 2001–2021 were retrieved for quantitative study. The data used in this paper are sourced from UN comtrade. The indexes (total population, GDP, solar power generation (GWh), renewable energy consumption as a percentage of end-use energy consumption, and total renewable energy generation (GWh)) used in the IRCA method to obtain the projection weight were collected on the World bank database.

## 4. Results of IRCA index method analysis

**Fig 4:**
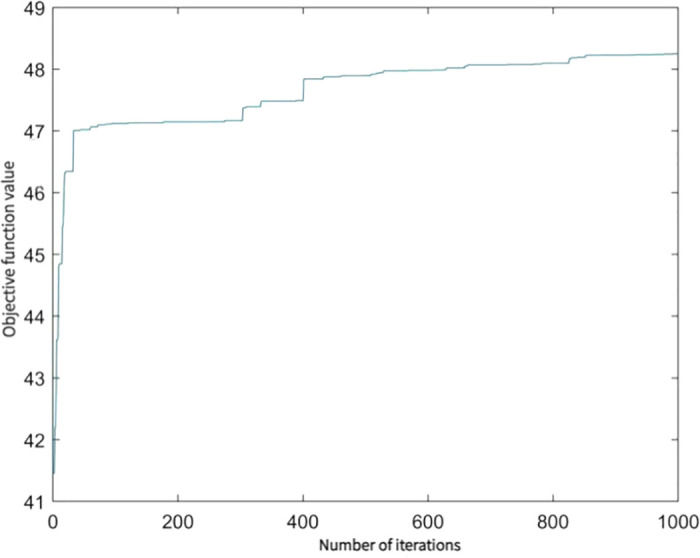
Genetic algorithm convergence process.

In this paper, the IRCA method is used to analyse the export trade competitiveness of solar photovoltaic products in CPTPP countries. To obtain the IRCA index for each commodity group in each year, this study uses a real number coded accelerated genetic algorithm(RAGA) to optimise the projection tracing method. We set the initial population size to 100, the maximum number of genetic generations to 1000, the crossover probability to 0.8 and the variation probability to 0.05, and the optimization process is carried out via MATLAB. According to the genetic algorithm, the final one-dimensional projection index is 48 and the weights of each variable are a = {0.0007, 0.9811, 0.0006, 0.1934, 0.0005} and the convergence process is shown in Fig 4. It can be seen that GDP (US$), solar power generation (GWh) and total renewable energy generation (GWh) all have relatively close weights and the most influential variable is population.

This study follows the traditional RCA index for classifying the level of competitiveness. The specific data are shown in Table 1.

**Table 1:** IRCA indices by commodity group in CPTTP countries.

Year	Australia		Mexico			Japan			Malaysia			Vietnam			Peru			Chile			Canada	
	B	C	A	B	C	A	B	C	A	B	C	A	B	C	A	B	C	A	B	C	B	C
**2001**	0.01	0.02	0.23	2.07	0.28	0.8	0.8	4.4	0.07	0.2	1.24	0.008	0.01	0.000	0.000	0.003	0.0002	0.014	0.002	0.0002	0.23	0.11
**2002**	0.01	0.05	0.34	1.93	0.22	0.8	0.8	5.1	0.04	0.13	1.05	0.015	0.02	0.000	0.005	0.006	0.0001	0.008	0.004	0.0000	0.27	0.1
**2003**	0.02	0.07	0.38	1.83	0.25	0.8	0.8	5.6	0.03	0.13	0.86	0.024	0.01	0.000	0.02	0.005	0.0036	0.007	0.006	0.0001	0.24	0.11
**2004**	0.01	0.08	0.34	1.47	0.27	1.1	0.8	5.6	0.05	0.14	0.8	0.003	0.01	0.000	0.017	0.011	0.0003	0.009	0.006	0.0002	0.26	0.07
**2005**	0.01	0.06	0.46	1.25	0.38	1.4	0.8	5.2	0.09	0.13	0.72	0.026	0.01	0.043	0.024	0.029	0.0001	0.02	0.007	0.0001	0.27	0.06
**2006**	0.01	0.06	0.47	1.13	0.47	1.1	0.8	4.6	0.03	0.13	0.71	0.231	0.02	0.062	0.016	0.003	0.0009	0.01	0.006	0.000	0.28	0.06
**2007**	0.01	0.06	0.51	0.82	0.34	0.4	0.7	3.8	0.04	0.12	0.53	0.335	0.01	0.055	0.021	0.002	0.0003	0.005	0.003	0.0001	0.26	0.06
**2008**	0.02	0.03	0.54	0.78	0.47	0.7	0.8	2.8	0.06	0.1	0.28	0.192	0.02	0.035	0.026	0.002	0.0002	0.016	0.004	0.0001	0.26	0.08
**2009**	0.01	0.01	0.51	0.62	0.72	1.2	0.7	2.5	0.13	0.09	0.34	0.079	0.01	0.008	0.032	0.006	0.0009	0.017	0.006	0.0002	0.27	0.05
**2010**	0.01	0.01	0.46	0.65	0.48	0.7	0.7	1.7	0.05	0.08	0.57	0.158	0.03	0.029	0.019	0.004	0.000	0.027	0.005	0.000	0.24	0.03
**2011**	0.01	0.01	0.56	0.72	0.64	0.8	0.9	1.9	0.08	0.09	0.63	0.065	0.06	0.041	0.02	0.002	0.000	0.005	0.007	0.000	0.25	0.04
**2012**	0.01	0.01	0.6	0.83	0.68	0.8	0.9	2.4	0.14	0.17	0.83	1.886	0.08	0.261	0.01	0.003	0.000	0.008	0.007	0.0001	0.25	0.08
**2013**	0.01	0.01	0.56	0.88	0.77	0.9	1	2.4	0.18	0.18	1.22	0.179	0.21	0.307	0.041	0.004	0.000	0.011	0.007	0.0002	0.24	0.06
**2014**	0.01	0.01	0.69	0.91	0.55	0.7	1	2.2	0.2	0.15	1.18	0.064	0.28	0.171	0.022	0.004	0.0008	0.002	0.005	0.0002	0.21	0.07
**2015**	0.02	0.01	0.57	0.93	0.72	0.6	0.9	1.8	0.24	0.14	1.35	0.09	0.56	0.776	0.02	0.003	0.0001	0.009	0.006	0.000	0.19	0.15
**2016**	0.02	0.01	0.45	1.07	0.74	0.7	0.9	1.8	0.29	0.15	1.72	0.13	0.73	2.12	0.03	0.003	0.0001	0.006	0.004	0.0164	0.21	0.15
**2017**	0.02	0.01	0.56	1.08	0.12	0.8	0.9	1.8	0.32	0.15	1.62	0.237	0.65	2.872	0.027	0.004	0.0001	0.006	0.017	0.0022	0.22	0.13
**2018**	0.02	0.00	0.08	1.08	0.10	0.08	0.99	1.76	0.02	0.17	1.59	0.31	0.62	2.20	0.03	0.0031	0.0001	0.0034	0.0034	0.0034	0.22	0.09
**2019**	0.02	0.00	0.06	1.12	0.10	0.10	1.26	1.85	0.02	0.21	1.67	0.19	0.79	3.36	0.03	0.0031	0.0001	0.0004	0.0004	0.0004	0.21	0.08
**2020**	0.02	0.00	0.05	1.08	0.07	0.08	1.35	1.90	0.02	0.25	1.37	0.14	0.94	3.55	0.03	0.0031	0.0001	0.0002	0.0002	0.0002	0.17	0.12
**2021**	0.03	0.00	0.07	1.03	0.05	0.09	1.38	1.87	0.03	0.24	1.31	0.18	1.24	3.30	0.02	0.0016	0.0002	0.0002	0.0002	0.0002	0.14	0.09

Source:Author’s calculations based on UN comtrade database data using CMS model, A, B and C for component products, power products, solar cells respectively.

(1) Japan: comparative advantages of most products show a falling and then rising trend

According to Table 1, the competitiveness of Japanese solar PV products is significantly higher than that of other CPTPP countries. For example, solar cells were at a strong level of comparative advantage during 2001–2004. However, from 2001–2017, the competitiveness level of solar cells had a more significant decline, from 5.6 in 2004 to 1.8 in 2017, with the comparative advantage falling from a very strong level to a sub-strong level. The comparative advantage of power products has been at a comparative disadvantage of below 1. Component products had a comparative advantage below 1 until 2004, when they crossed over to generate an average advantage. However, after 2007, there was stability below 1, indicating a less significant comparative advantage for this product. This leads to the conclusion that the reduction of solar cells’ comparative advantage was the primary cause of Japan’s overall competitiveness between 2012 and 2017. Except for module products, the comparative advantage of the rest of the products rose in 2018–2021, indicating that the overall comparative advantage of Japanese PV products is on an upward trend.

(2) Australia: export competitiveness is volatile and at a low level

The comparative advantage solar cells decreased from a high of 0.08 in 2004 to 0.01 in 2010, and it also fluctuated at a low level from 2010 and 2021, reaching just 0.004 in 2021. This suggests that Australia’s export competitiveness is extremely poor and unstable for this product. The competitive advantage of power products has also been weak, ranging between 0.01 and 0.02 with a comparatively constant variation. It is evident that Australia’s exports are becoming less competitive overall, i.e., the changes in product share and structure are not well suited to changes in global market demand.

(3) Canada: overall comparative advantage is low and less volatile

The comparative advantage of power supply products was at a weaker level from 2001–2009, but on an upward trend. After 2009, it started to decline again and the level of competitiveness in 2021 is 0.09% lower than in 2001. It indicates that this product’s level of competitiveness has been lower. The solar cell industry’s competitiveness fluctuates, first declining and then rising. Prior to resuming to grow once more and recovering to 0.09 in 2021, it dropped from 0.11 in 2001 to 0.03 in 2010. Power goods have always had a bigger comparative advantage than solar cells, despite the two items’ comparative advantages being at a lower level than they once were. Due to the interaction of changes in the competitiveness of the two goods, the overall competitiveness of Canadian solar PV products is increasing.

(4) Malaysia: Comparative advantage is generally low, but changes vary from product to product

In Malaysia, solar PV products have a growing overall comparative advantage. Component and power product comparative advantages exhibit a tendency of initially falling and then increasing. It is clear that after reaching its lowest point in 2009, solar cells’ competitiveness began to increase as the economy recovered. From 0.34 in 2009 to 1.31 in 2021, it increased from a weaker to a stronger level. Malaysia’s export share for this product is not effectively adapted to changes in global market demand, despite the fact that the competitiveness of power supply items has increased and their quantity has decreased. Even though it was constantly lower, the competitive advantage of component products exhibited a general rising tendency, dropping from 0.07 in 2001 to 0.0.03 in 2021.

(5) Mexico: comparative advantage declined from the second strongest to the medium level, with a small decline in overall competitiveness

Although there was a significant decline in its comparative advantage in 2008–2009, it swiftly recovered to a medium level state as the economy recovered, placing power supply products at the next strongest level. Two items’ comparative advantages—component goods and solar cells—have been at a lower level. Solar cells’ comparative advantage has recently declined significantly since 2016, whereas component products’ comparative advantage has marginally increased. The combined impact of the three items resulted in a small drop in the competitiveness of Mexican exports. It demonstrates how successfully Mexico solar PV exports have been able to adjust to changes in global market demand in recent years, both in terms of share and structure.

(6) Peru: competitiveness at a low level and eventually declining

In 2010 and 2011, the component product’s comparative advantage drastically decreased. The comparative advantage recovered quickly in 2010 as the economy expanded, but it dropped once more in 2016, demonstrating the product’s poor and erratic competitiveness in recent years. Power products’ comparative advantage has been at a lower level; it spiked in 2005 and helped Peru’s export growth, but it quickly dropped from 0.029 to 0.003 in 2006 before stabilizing at 0.001, showing that the product’s competitiveness is extremely low and contributes very little to export growth. Solar cells typically have a modest comparative advantage, which has been varying between 0.0001 and 0.0003. So, it may be inferred that the situation is not hopeful and that Peru’s exports of solar photovoltaic products are less competitive.

(7) Vietnam: sharp increase in comparative advantage of individual products and an overall upward trend

The comparative advantage of components had a surge in 2012, reaching 1.886, then started to fall, but started to rise again after 2014, and although the comparative advantage was still low in 2017, a steady upward trend in competitiveness can be seen after 2014. The comparative advantage of power supply products has been weak from 2001 to 2012, but has been rising and can be seen to have reached 1.24 in 2021, a significant increase compared to 0.02 in 2002. In addition, the comparative advantage of solar cells rose sharply after 2014 and is in an optimistic situation, reaching 3.30 in 2021, implying that at this time Vietnam has a strong export competitiveness for this product. With the combined effect of the three products, the competitiveness of Vietnamese solar PV products shows a good upward trend.

(8) Chile: overall competitiveness is weak but on an upward trend

Comparative advantage of component goods experienced a significant increase in 2011 before declining and eventually showing a stable downward trend. Power supply goods’ comparative advantage has been at a lower level, but it significantly increased between 2016 and 2017. Moreover, between 2001 and 2014, the comparative advantage of solar cells varied between 0.001 and 0.002, but it increased significantly in 2015 before declining once more in 2016. However, compared with the comparative advantage before 2015, the overall comparative advantage in 2021 remains large. It is easy to see that the overall competitiveness of Chilean exports of solar PV products has risen, but is less competitive, so the country needs to focus on improving the competitiveness of its own products in its initiatives to increase export growth.

## 5. Analysis of CMS model results

**Fig 5:**
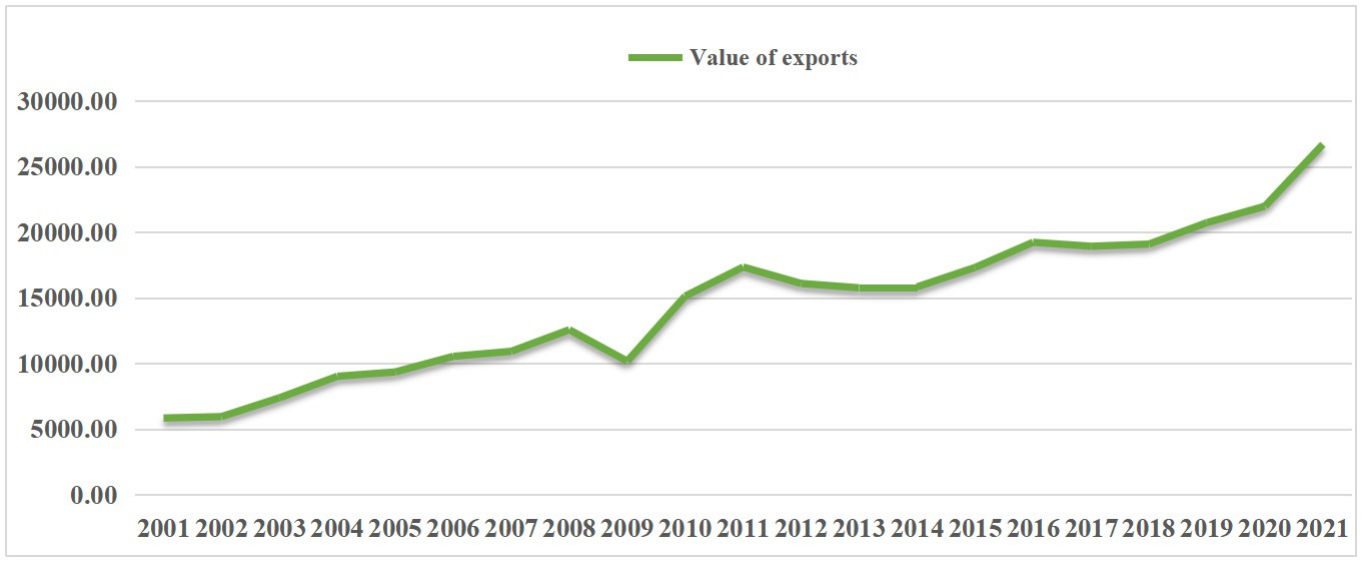
Change in solar PV product exports from CPTPP countries, 2001–2017 (US$ million).

As can be seen from Fig 5, the five stages of the trade trend for solar PV products from CPTPP nations are as follows: The export value of solar PV products in CPTPP countries generally increased between 2001 and 2007 before the financial crisis began; however, as a result of the effects of the global financial crisis, this pattern significantly changed between 2008 and 2009. The export value of solar PV products from CPTPP nations climbed and decreased by a lesser margin from 2012 to 2017, demonstrating a fluctuating tendency. The export value of solar PV products from CPTPP countries began to rebound in 2010 and 2011 as the global economy started to recover. Period 2018–2021 contains the entry into force of the CPTPP treaty and the outbreak of the new crown epidemic.

Based on the above stages, we used the CMS model to measure the export data of solar photovoltaic products of each CPTPP country at each stage, and then compared the change trend of each interval in order to reveal the change pattern of the export competitiveness of solar photovoltaic products of each CPTPP country.

### (1) Japan

Exports grew by US$3,958.7 million in Phase 1 and US$68 million in Phase 3, the lower growth being due to the slow recovery of the world economy after the recent financial crisis. The specific data of CMS model is shown in Table 2.

**Table 2:** Results of CMS model decomposition of Japan’s solar PV export growth (US$ million).

Year	2001–2007		2008–2009		2010–2011		2012–2017		2018–2021	
	Amount of contribution	Contribution rate	Amount of contribution	Contribution rate	Amount of contribution	Contribution rate	Amount of contribution	Contribution rate	Amount of contribution	Contribution rate
Growth in export value	3958.69	100.00%	-1784.54	-100.00%	668.81	100.00%	-1870.14	-100.00%	470.25	100.00%
Structural effects	6665.06	168.37%	-873.54	-48.95%	257.77	38.54%	42.38	2.27%	2157.93	458.89%
Product effects	1902.09	48.05%	184.08	10.32%	-5.60	-0.84%	0.97	0.05%	105.51	22.44%
Growth effects	4762.97	120.32%	-1057.63	-59.27%	263.38	39.38%	41.41	2.21%	2052.42	436.46%
Competitive effects	-839.26	-21.20%	-989.42	-55.44%	398.01	59.51%	-1899.81	-101.59%	-1236.04	-262.85%
Overall competitiveness effect	-34.64	-0.88%	-839.25	-47.03%	392.83	58.74%	-1901.67	-101.69%	-1196.48	-254.44%
Specific competitive effects	-804.62	-20.33%	-150.18	-8.42%	5.18	0.78%	1.85	0.10%	-39.56	-8.41%
Second order effects	-1867.11	-47.16%	78.43	4.39%	13.02	1.95%	-12.71	-0.68%	-451.64	-96.04%
Pure Second Order	-1363.16	-34.43%	132.44	7.42%	12.77	1.91%	-9.87	-0.53%	-398.45	-84.73%
Dynamic second order	-503.95	-12.73%	-54.01	-3.03%	0.25	0.04%	-2.84	-0.15%	-53.20	-11.31%

Source: Authors’ calculations based on UN comtrade database data using CMS model.

#### ① Competitiveness effect is the main source inhibiting the growth of Japanese solar PV exports in all phases

The competitiveness effect’s contribution to Japan’s exports increased steadily after turning from negative to positive in 2008–2009. The competitiveness effect rose dramatically once more to 101.59% in the fourth period as a result of the global economic recovery, demonstrating that increasing competitiveness is the main driving force behind the expansion of Japan’s PV product exports.

Four of the five phases of overall competition from 2001–2021 are negative, indicating that the increase in overall competitiveness had a negative effect on export growth in both of the first two phases. The contribution rises to a positive value in the third period before falling to -262.85% in the fifth period, suggesting that this effect is increasingly becoming the main force inhibiting the growth of Japanese solar PV exports.

The specific competitive effects were negative in 2001–2007 and 2008–2009, positive in the two phases 2010–2011 and 2012–2017, and increased slightly in the fourth phase compared to the third phase. This indicates that the structure of Japanese PV products has been optimised and adapted to changes in international market demand. But in the fifth stage its again had a dampening effect on export growth.

#### ② Structural effects down and then back, world import demand pulls on Japanese exports

The structural effect shows a continuous down and then back trend, and the growth effect, which accounts for the majority of the structural effect, is also declining sharply and then rising. The decline from 120.32% in 2001–2007 to -2.21% in 2012–2017 may be explained by the fact that the export market for Japanese PV products is increasingly concentrated in regions with low import demand. But the structural effect rises to 458.89% in 2018–2021.

The value of exported Japanese solar PV products decreased by US$1,870 million during the fourth period. The competitiveness effect continued to be negative, but at this point, because of the favorable growth effect, it finally caused a decrease in exports because its absolute value was lower than the competitiveness effect’s absolute value. In the fifth period, Japan’s solar PV exports increased by USD 47,245 million. Although the competitiveness effect was negative, the export value eventually kept growing because the growth effect was positive and outweighed the competitiveness effect.

#### ③ The second-order effect gradually becomes negative and shows a continuous downward trend, with a decreasing effect on export growth

The second-order effect in the second stage was positive and helped to increase the export value of Japanese solar PV products, although the contribution was only -4.39% due to a combination of pure and dynamic second-order effects. Both the first and third stages of the second-order impact were negative, showing that the second-order effect operated as a backward drag on the increase of exports of Japanese solar PV products in both periods.

With the exception of the first stage at 47.6%, the second-order effects are very small and show a decreasing trend in the remaining three stages. Although the second-order effect of the fifth stage reaches -96.04%, its effect on export growth is smaller relative to the structural and competitiveness effects. It shows that the interaction of market structure and competitiveness is playing an increasingly smaller role in the growth of Japanese solar PV exports.

### (2) Australia

Exports grew by US$119.145 million in Phase I and US$55.418 million in Phase III, the lower growth being due to the slow recovery of the world economy after the recent financial crisis. The specific data of CMS model is shown in Table 3.

**Table 3:** Results of the CMS model decomposition of Australia’s solar PV export growth (US$ million).

Year	2001–2007		2008–2009		2010–2011		2012–2017		2018–2021	
	Amount of contribution	Contribution rate	Amount of contribution	Contribution rate	Amount of contribution	Contribution rate	Amount of contribution	Contribution rate	Amount of contribution	Contribution rate
Growth in export value	119.145	100.00%	-96.317	100.00%	55.418	100.00%	24.081	100.00%	35.102	100.00%
Structural effects	34.768	29.18%	-18.562	19.27%	1.561	2.82%	0.113	0.47%	37.411	106.58%
Product effects	7.891	6.62%	3.166	-3.29%	-0.115	-0.21%	-0.249	-1.03%	1.177	3.35%
Growth effects	26.877	22.56%	-21.729	22.56%	1.676	3.02%	0.362	1.50%	36.233	103.22%
Competitive effects	25.565	21.46%	-86.291	89.59%	52.294	94.36%	23.972	99.55%	-1.653	-4.71%
Overall competitiveness effect	35.160	29.51%	-86.115	89.41%	52.071	93.96%	23.597	97.99%	-0.855	-2.44%
Specific competitive effects	-9.595	-8.05%	-0.176	0.18%	0.223	0.40%	0.375	1.56%	-0.797	-2.27%
Second order effects	58.811	49.36%	8.537	-8.86%	1.563	2.82%	-0.003	-0.01%	-0.656	-1.87%
Pure second-order effects	41.524	34.85%	11.550	-11.99%	1.678	3.03%	0.125	0.52%	-0.533	-1.52%
Dynamic second order	17.288	14.51%	-3.014	3.13%	-1.455	-2.63%	-0.128	-0.53%	-0.123	-0.35%

Source: Authors’ calculations based on UN comtrade database data using CMS model.

#### ① Structural utility has a greater positive contribution to export value growth

From 29.18 per cent in Stage 1 to 19.27 per cent in Stage 2, it has fallen to 0.47 per cent in Stage 4, indicating that world import demand is becoming less influential in the growth of Australian exports. It is worth noting that when export volumes grow, there is always a positive contribution from structural utility, although this contribution is declining. However, the structural effect of the fifth stage contributed 106.58% to export growth, indicating that world import demand is having an increasing impact on Australian export growth.

Growth effects account for a large share of structural effects, with a growth effect of 22.56% in phases 1 and 2, and a reduction in the growth effect to 3.02% in phase 3. It can be seen that the rise in Australian exports as a result of rising global imports first stayed stable, then abruptly decreased. The international financial crisis’ delayed economic recovery in Australia’s top export markets and the drop in demand for solar PV products are likely to blame for this.

#### ② Improved competitiveness is the primary pulling force behind the growth of its PV exports, but competitiveness is on a downward trend

The competitiveness effect can be seen to increase sharply and remain steadily rising from stage 2 onwards, with a contribution of 89% in stage 2, 94% in stage 3 and 99% in stage 4, thus showing that the increased competitiveness of Australian solar PV products is the primary driver of its PV export growth. However, the fifth stage has a dampening effect of 4.71% on export growth due to the decline in competitiveness.

Overall competitiveness accounted for about 99% of the competitiveness effect and maintained that share regardless of whether the competitiveness effect was formal or negative, outweighing the specific competition effect in all phases. The specific competitiveness effect is negative at -8.05%, -0.18% and -4.71% in stages 1, 2 and 5 respectively, indicating that the export structure is not well adapted to changes in international market demand. The third and fourth stages became positive.

#### ③Secondary effects have contributed to Australia’s export growth, but are diminishing

The absolute value of the second-order effect became smaller and smaller, falling from 49.36% in the first stage to -1.87% in the fifth stage, meaning that the interaction between the structural effect and the competitiveness effect had a decreasing impact on export growth. The secondary effects of the first four stages, implying that the second-order effect plays a role in promoting export growth, but due to the financial crisis experienced at this time, the global economic downturn has caused a significant decline in competitiveness, resulting in a reduction in exports.

With the exception of the fifth stage, the pure second-order effect both play a positive effect on export growth, indicating that the changes in Australia’s export structure were well adapted to changes in world demand. The pure second-order effect is -1.52% for the fifth stage, indicating that although export volumes were rising in 2008–2009, the structure of Australia’s exports was still deteriorating and had a negative effect on export growth.

### (3) Peru

Exports increased by US$13,1 million during the first phase, US$10,2 million during the second, and US$11,7 million during the third. The slow global economic recovery following the financial crisis and the challenges experienced by Peruvian solar PV exports were the main causes of the decline in exports. The fifth period saw a negative growth of $1.04 million. This indicates that the export value of solar photovoltaic products fluctuates more frequently. The specific data of CMS model is shown in Table 4.

**Table 4:** Results of the CMS model decomposition of Peru’s solar PV export growth (US$ million).

Year	2001–2007		2008–2009		2010–2011		2012–2017		2018–2021	
	Amount of contribution	Contribution rate	Amount of contribution	Contribution rate	Amount of contribution	Contribution rate	Amount of contribution	Contribution rate	Amount of contribution	Contribution rate
Growth in export value	13.19	100.00%	10.17	100.00%	-1.17	100.00%	14.15	100.00%	-1.04	100.00%
Structural effects	1.64	12.40%	-3.44	-33.80%	1.12	95.97%	0.31	2.16%	0.42	-40.21%
Product effects	-0.6	-4.58%	-1.25	-12.25%	0.46	39.62%	0.2	1.42%	-0.53	51.31%
Growth effects	2.24	16.98%	-2.19	-21.55%	0.66	56.35%	0.1	0.74%	0.95	-91.52%
Competitive effects	4.79	36.30%	16.46	161.84%	-2.31	-197.69%	13.51	95.42%	-1.27	122.15%
Overall competitive effect	4.17	31.64%	14.27	140.33%	-1.77	-151.49%	13.98	98.75%	-1.51	144.84%
Specific competitive effects	0.61	4.66%	2.19	21.50%	-0.54	-46.21%	-0.47	-3.33%	0.24	-22.68%
Second order effects	6.76	51.30%	-2.85	-28.03%	0.02	1.72%	0.34	2.43%	-0.19	18.06%
Pure second-order effects	7.77	58.96%	-2.2	-21.66%	-0.07	-6.34%	0.07	0.50%	-0.41	39.38%
Dynamic second-order effects	-1.01	-7.66%	-0.65	-6.37%	0.09	8.07%	0.27	1.93%	0.22	-21.31%

Source: Authors’ calculations based on UN comtrade database data using CMS model.

#### ①Early export volume growth was positively impacted by structural utility, but later export growth was restrained

From 12.4% in Stage 1 to -33.8% in Stage 2, it has risen to 2,16% in Stage 4, indicating that world import demand is becoming less influential in the growth of Australian exports. It is worth noting that when export volumes grow, there is always a positive contribution from structural utility, albeit a declining one. However, the structural effect of the fifth stage contributes -40.21% to export growth, indicating that the change in world import demand inhibits the export of PV products.

The growth effect on export growth changes more frequently, indicating that the role played by the increase in world demand on Peruvian exports is more unstable. The growth effect decreases from 22.56% in the first stage to -91.52% in the fifth stage, this is probably due to the slow economic recovery in Australia’s main export markets following the international financial crisis and the decline in demand for solar PV products.

#### ②The expansion of its PV exports is mostly being driven by increased competition, with overall competitiveness contributing more heavily than specific competitive effects

The competitiveness effect can be seen to increase sharply, the contribution of the latter four stages to export growth is greater than 90% in absolute terms, and in addition, the competitiveness effect moves in the same direction as export growth, regardless of whether exports are growing or declining. thus showing that the increased competitiveness of Peru’s solar PV products is the primary driver of its PV export growth.

Overall competitiveness accounted for about 99% of the competitiveness effect and maintained that share regardless of whether the competitiveness effect was formal or negative, outweighing the specific competition effect in all phases. The specific competitiveness effect is positive but the last three phases are negative indicating that the export structure is not well adapted to changes in international market demand.

#### ③ Secondary effects have contributed to Peru’s export growth, but are diminishing

The absolute value of the second-order effect became smaller and smaller, falling from 51.30% in the first stage to -18.06% in the last stage, and to 0.01% in the fourth stage, meaning that the interaction between the structural effect and the competitiveness effect had a decreasing impact on export growth. The second stage, however, has the biggest dampening impact on export growth because of the financial crisis that was prevalent at the time, which caused a major fall in competitiveness, which in turn caused a decrease in final exports.

### (4) Malaysia

All four stages showed an increase in exports, with the first phase increasing by $275 million, the second by $52 million, and the third by $161 million. The fifth phase added $819 million. The reason for the low export growth in the second and third stages is that the global economic recovery after the financial crisis has slowed down the export of Malaysia’s solar photovoltaic products. The specific data of CMS model is shown in Table 5.

**Table 5:** Results of the CMS model decomposition of Malaysia’s solar PV export growth (US$ million).

Year	2001–2007		2008–2009		2010–2011		2012–2017		2018–2021	
	Amount of contribution	Contribution rate	Amount of contribution	Contribution rate	Amount of contribution	Contribution rate	Amount of contribution	Contribution rate	Amount of contribution	Contribution rate
Growth in export value	275	100.00%	52	100.00%	161	100.00%	1475	100.00%	819.22	100.00%
Structural effects	22.26	808.81%	-1.14	-221.28%	0.86	53.51%	0.16	1.08%	1795.05	219.12%
Product effects	6.79	246.80%	0.21	40.62%	-0.05	-3.17%	0.00	0.00%	162.69	19.86%
Growth effects	15.46	562.01%	-1.35	-2.619030608	0.92	56.68%	0.16	1.07%	1632.37	199.26%
Competitive effects	-5.72	-208.04%	1.86	361.02%	0.72	44.82%	14.50	98.27%	-693.20	-84.62%
Overall competitiveness effect	-4.84	-176.06%	2.15	417.83%	0.68	41.97%	14.52	98.41%	-614.92	-75.06%
Specific competitive effects	-0.88	-31.98%	-0.29	-56.81%	0.05	2.84%	-0.02	-0.15%	-78.28	-9.56%
Second order effects	-13.78	-500.78%	-0.20	-39.74%	0.03	1.67%	0.10	0.66%	-282.63	-34.50%
Pure Second Order	-9.30	-337.90%	-0.25	-48.32%	0.02	1.44%	0.08	0.51%	-223.46	-27.28%
Dynamic second order	-4.48	-162.88%	0.04	8.59%	0.00	0.24%	0.02	0.15%	-59.17	-7.22%

Source: Authors’ calculations based on UN comtrade database data using CMS model

#### ① Structure effect is still the main driver, the effect of growth effect outperforms the effect of product effect

The structural effect reached 808.81% in the first stage, and decreased to the minimum 221.28% in the second stage, The fifth stage rose to 219.12%. Due to the outbreak of the financial crisis, the world’s total import of solar photovoltaic products decreased, which played a reverse pulling effect on Malaysia’s export growth. In recent years, due to the importance of the country as well as the growth of world demand, have contributed in varying degrees to the growth of exports.

The growth effect in the first and third phases was 562.01% and 56.68%, respectively, showing that these two stages’ rapid rises in the demand for solar photovoltaic products on the worldwide market contributed to Malaysia’s export growth to some extent.

Despite the fact that 2008–2009 saw a financial crisis and a severe decline in global import demand, the product effect was still 40.62%, showing that it significantly boosted Malaysia’s export growth during this time. The contribution of product effect to export growth in 2010–2011 was -3.17%, acting as a reverse pulling force, showing that Malaysia’s export products failed to meet market demand at the appropriate moment during this period.

#### ② The competitiveness effect is fluctuating, with the overall competitiveness gradually increasing its impact on export growth

The contribution rate of competitiveness effect to export growth was the lowest in 2001–2007, at -208.04%. It had a sharp increase in 2008–2009, decreased in 2010–2011, and finally reached -84.62% in 2018–2021. It can be seen that the competitiveness of Malaysia’s solar PV products is fluctuating

The contribution of the overall competition effect in the four periods is in the same direction as the competitiveness effect, and the contribution rate is better than the specific competition effect. It is worth noting that the contribution rate of the overall competition effect reached -75.06% in the 2018–2021 period, which means that the overall competitiveness of Malaysia’s photovoltaic products has gradually become the primary driving force for Malaysia’s export growth.

Aside from the third stage, the special competition effect has a negative influence. We can see that the export structure of Malaysia’s photovoltaic products does not fit well with the changes in the import demand of the international market when combined with the idea of the unique competitive effect.

#### ③ The second-order effect on export growth becomes progressively weaker

The second-order effect had a contribution of -500.78% from 2001 to 2007, which indicates that this effect significantly hampered Malaysia’s export growth. The dynamic second-order effect was -162.88%, while the pure second-order effect had a value of 337.9%. In other words, Malaysia’s solar photovoltaic products became less competitive throughout this time, and the country’s export structure did not adapt adequately to the shifts in global import demand.

The second-order effect of the fifth stage is -34.50%, higher than the first stage of -500.78%. The competitiveness of Malaysia’s solar photovoltaic products increased during this time, and its export structure was more in line with changes in import demand on the global market, which gradually encouraged the growth of Malaysia’s export. Dynamic second-order effect and pure second-order effect both increased.

### (5) Canada

Phase I saw an increase in exports of $188.7 million, Phase II a decrease of 167.58 million, Phase III a rise of 49.02 million, and Phase IV a rise of 11.2 million. Exports expanded more in Phase III than in Phase IV, although at a slower rate than in Phase III due to the gradual recovery of the world economy. The new crown outbreak may also have an impact on the fifth phase, which would lead to a sharp decline in exports. The specific data of CMS model is shown in Table 6.

**Table 6:** Results of the CMS model decomposition of Canada’s solar PV export growth(US$ million).

Year	2001–2007		2008–2009		2010–2011		2012–2017		2018–2021	
	Amount of contribution	Contribution rate	Amount of contribution	Contribution rate	Amount of contribution	Contribution rate	Amount of contribution	Contribution rate	Amount of contribution	Contribution rate
Growth in export value	188.70	100.00%	-167.58	100.00%	49.02	100.00%	11.28	100.00%	-79.09	100.00%
Structural effects	519.95	275.54%	-91.37	54.52%	16.92	34.51%	0.98	8.69%	212.98	-269.30%
Product effects	-64.96	-34.43%	-6.11	3.65%	-1.25	-2.54%	-2.41	-21.35%	9.64	-12.19%
Growth effects	584.91	309.96%	-85.26	50.88%	18.17	37.05%	3.39	30.04%	203.34	-257.11%
Competitive effects	-125.13	-66.31%	-85.44	50.99%	31.17	63.59%	9.88	87.55%	-219.13	277.08%
Overall competitiveness effect	-150.98	-80.01%	-95.04	56.72%	29.90	60.99%	7.85	69.60%	-213.58	270.06%
Specific competitive effects	25.85	13.70%	9.60	-5.73%	1.27	2.60%	2.03	17.95%	-5.56	7.03%
Second order effects	-206.12	-109.23%	9.23	-5.51%	0.93	1.90%	0.42	3.76%	-72.93	92.22%
Pure Second Order	-203.23	-107.70%	11.44	-6.82%	1.00	2.04%	0.05	0.45%	-70.64	89.32%
Dynamic second order	-2.88	-1.53%	-2.21	1.32%	-0.07	-0.14%	0.37	3.31%	-2.29	2.90%

Source: Authors’ calculations based on UN comtrade database data using CMS model.

#### ① The structural effect on export growth is gradually decreasing, and exports are more dependent on import demand

Between 2001 and 2007, the proportion of global imports that changed was responsible for 275.54% of the rise in Canadian exports. It is also simple to observe that during this time, the growth impact even outweighed the structural effect, with the excess only balancing the detrimental effects of the product effect, which was the primary factor driving export growth at the time.

The growth impact and the product effect were -50.88% and -3.65%, respectively, however the structural effect contributed 54.52% to export growth in 2008–2009, suggesting that the structure of Canada’s own exports did not change to reflect changes in market demand. As the economy recovered, the growth effect contributed 30.04% from 2012–2017, but the fifth stage growth effect contributes 257.11% to export growth.

#### ② Competitiveness effect has improved and is becoming the main determinant of export growth

The contribution of the competitiveness effect was negative in both phases I and II, at -66.31% and -50.99% respectively, meaning that the competitiveness of export products declined in both phases to the extent that it hindered export growth. The third period saw an increase from 63.59% to 87.55% in the fourth period, indicating that the competitiveness of PV products has improved in recent years and has contributed significantly to the growth of Canadian exports. But the fifth stage and the role of exports -277.08%, indicating that the competitiveness of Canadian photovoltaic products are more negatively affected by the new crown epidemic.

In all five phases, the aggregate competition effect contributed more than any individual competition effect. The deterioration in the overall competitiveness of Canadian products from 2001 to 2007 hampered export development, and the overall competition effect was negative in the first period. However, the specific competition effect was favorable in the second period, supporting export growth.

The competitiveness of Canadian PV goods has risen to 69.6% in recent years, suggesting that competitiveness has taken over as the main engine of Canadian export growth in recent years. Both the 2010–2011 and 2012–2017 periods would be favorable for both overall and particular competitiveness.

#### ③Although the export product mix adapts gradually to changes in consumer demand, export growth is still constrained

The second-order effect from 2001 to 2007 was -109.23%, with the pure second-order utility having a dampening effect of -107.70% on the growth of Canadian PV exports. This shows that the change in the share of Canadian exports was significantly out of step with the change in the demand for PV imports on the international market.

In 2008–2009, the second-order effect was positive, but it was only 5.51% in absolute terms. This was primarily because of the favorable pure second-order effect. The proportion of Canadian exports began to alter in response to shifts in global demand, but the structural and competitiveness effects were unfavorable and significant in absolute terms, which led to a decline in final exports. The second-order effect’s contribution for the period of 2018 to 2021 is -92.22%, which is mostly attributable to the fall in demand from nations affected by the new crown epidemic and the difficulty of conducting export commerce as a result of anti-epidemic measures taken by some nations.

For the period 2012–2017, both the secondary effect and its dichotomous effect were positive. This suggests that the interaction between the increased competitiveness of PV products and the gradual adaptation of the structure of Canada’s own exports to changes in market demand has contributed to the growth of Canadian PV exports.

### (6) Singapore

With the exception of Phase II, all showed an increasing trend. Exports grew by US$706 million in Phase 1, US$909 million in Phase 3 and US$758 million in Phase 4. The negative growth in exports in Phase 2 was due to the financial crisis that hit Singapore’s solar PV exports at this time. The specific data of CMS model is shown in Table 7.

**Table 7:** Results of CMS model decomposition of Singapore’s solar PV export growth(US$ million).

Year	2001–2007		2008–2009		2010–2011		2012–2017		2018–2021	
	Amount of contribution	Contribution rate	Amount of contribution	Contribution rate	Amount of contribution	Contribution rate	Amount of contribution	Contribution rate	Amount of contribution	Contribution rate
Growth in export value	706.36	100.00%	-349.86	100.00%	909.89	100.00%	757.78	100.00%	571.44	100.00%
Structural effects	952.4	134.83%	-241.58	-69.05%	65.85	7.24%	15.09	1.99%	913.46	159.85%
Product effects	66.28	9.38%	-1.34	-0.38%	-0.79	-0.09%	2.47	0.33%	36.42	6.37%
Growth effects	886.12	125.45%	-240.24	-68.67%	66.64	7.32%	12.63	1.67%	877.04	153.48%
Competitive effects	-70.51	-9.98%	-125.43	-35.85%	817.52	89.85%	741.69	97.88%	-243.64	-42.64%
Overall competitive effect	-68.5	-9.70%	-126.55	-36.17%	817.04	89.79%	741.30	97.83%	-231.11	-40.44%
Specific competitive effects	-2.01	-0.28%	1.12	0.32%	0.49	0.05%	39	0.05%	-12.53	-2.19%
Second order effects	-175.54	-24.85%	17.15	4.90%	26.52	2.91%	1	0.13%	-98.39	-17.22%
Pure second-order effects	-114.52	-16.21%	16.79	4.80%	26.23	2.88%	3.85	0.51%	-78.54	-13.74%
Dynamic second-order effects	-61.01	-8.64%	0.36	0.10%	0.29	0.03%	-286	-0.38%	-19.85	-3.47%

Source: Authors’ calculations based on UN comtrade database data using CMS model.

#### ① Structural effects of falling and then rising, and the influence of growth effect is larger than that of product effect

From 134.83 percent in the first stage to 69.05 percent in the second stage, a drop of about 50 percent, and 90 percent from the second to the third stage. The increase in global demand leading up to 2017 did not significantly enhance Singapore’s exports. The growth in exports was mostly brought on by Singapore’s solar PV goods becoming more competitive. However, the structural effect during the period of 2018–2021 contributes 159.85% to export growth, demonstrating that the main driver of export growth during this time is global demand.

Each stage’s growth effect outperforms the product effect and makes up a sizable amount of the structure effect. The growth effect’s first-stage contribution rate to the export of solar products was 125.45%. The structural impact, growth effect, and product effect were all negative in 2008–2009, which meant that as the financial crisis began, not only did the global demand for solar products decline, but also the export structure of Singapore’s photovoltaic products deteriorated.

#### ② Overall competitiveness in competitiveness effect greatly promotes export growth

The competitiveness effect decreased from -9.98% in 2001–2007 to -35.85% in 2008–2009, primarily as a result of Singapore’s exports’ dramatic loss in competitiveness as a result of the financial crisis, which also hampered export growth. Yet from 2010 to 2011, the competitiveness impact started to rise, and from 2012 to 2017, it surged to 97.88%. It is clear that the increased competitiveness of Singapore’s solar products has significantly increased their export in recent years.

The overall competitiveness of PV products in Singapore has significantly increased to support the export of PV products in Singapore because the overall competition effect is superior to the specific competition effect. Yet, the impact of specialized rivalry on export growth is minimal, and even the impact from 2012 to 2017 was essentially nonexistent. However, due to the epidemic, the competitiveness of Singapore PV products decreases and depresses export growth in 2018–2021.

#### ③ Second-order impacts increase and subsequently decrease their contribution to export growth, finally having a dampening effect

The contribution rate of the second-order effect between 2001 and 2007 is -24.85%, and the negative value denotes the inhibitory effect on export growth. Of this, the contribution rate of the pure second-order effect has a 16.21% inhibitory effect on export growth, indicating that the change in Singapore’s export product share does not adapt to the change in global market demand. The contribution rate is slightly lower but the second-order effects of the final three stages are all positive, indicating that the interaction between rising competitiveness and adapting export structures to shifting global market demands positively influences export growth, but this promotion effect is waning. This effect decreases to -17.22% in the fifth stage.

### (7) Vietnam

Among the four stages of export growth, the rest showed a trend of growth except for the second stage. The specific data of CMS model is shown in Table 8.

**Table 8:** Results of CMS model decomposition of Vietnam’s solar PV export growth(US$ million).

Year	2001–2007		2008–2009		2010–2011		2012–2017		2018–2021	
	Amount of contribution	Contribution rate	Amount of contribution	Contribution rate	Amount of contribution	Contribution rate	Amount of contribution	Contribution rate	Amount of contribution	Contribution rate
Growth in export value	19.03	100.00%	-10.89	100.00%	16.69	100.00%	2641.23	100.00%	5462.09	100.00%
Structural effects	0.13	0.68%	-2.88	-26.45%	1.15	6.91%	5.61	0.21%	909.11	16.64%
Product effects	-0.03	-0.13%	-0.44	-4.01%	0.28	1.67%	4.34	0.16%	71.93	1.32%
Growth effects	0.15	0.81%	-2.44	-22.44%	0.88	5.25%	1.27	0.05%	837.18	15.33%
Competitive effects	6.8	35.74%	-9.37	-86.08%	15.25	91.40%	2627.92	99.50%	3387.89	62.03%
Overall competitive effect	7.19	37.80%	-9.75	-89.55%	15.32	91.81%	2626.32	99.44%	3497.48	64.03%
Specific competitive effects	-0.39	-2.06%	0.38	3.47%	-0.07	-0.41%	1.6	0.06%	-109.59	-2.01%
Second order effects	12.1	63.59%	1.36	12.52%	0.28	1.69%	7.71	0.29%	1165.10	21.33%
Pure second-order effects	11.04	58.04%	1.25	11.52%	0.49	2.93%	13.66	0.52%	1092.11	19.99%
Dynamic second-order effects	1.05	5.54%	0.11	1.00%	-0.21	-1.25%	-5.95	-0.23%	72.99	1.34%

Source: Authors’ calculations based on UN comtrade database data using CMS model.

#### ① Demand for Vietnamese PV products in the world market is not increasing at the same rate as exports

The structural effect supported the increase of Vietnam’s PV product exports by 0.68% between 2001 and 2007. It is not difficult to see that during this time, the growth effect was 0.81% and the product effect was -0.13%, i.e., the main driver of export growth during this time was global market demand, while PV products exported by Vietnam themselves saw a decline in quality and failed to satisfactorily meet the needs of the market, leading to the negative product effect.

The financial crisis had a negative structural impact in 2008–2009, which contributed to a 26.45% dampening effect on export growth. At the same time, there was a fall in demand for Vietnamese PV products on the global market, which also had a dampening effect on Vietnamese exports. The structural effect soon rose again from 6.91% in the third phase to 16.64% in the fifth phase, contributing significantly to export growth.

#### ② Quality improvement makes products more competitive and the competitiveness effect has an upward trend

The competitiveness effect in the first phase contributed 35.74% to export growth, but in 2008–2009 it decreased to -86.06%, showing that Vietnam was significantly negatively impacted by the financial crisis. From 2010 to 2011, Vietnamese products’ competitiveness increased significantly, almost serving as the primary engine for the expansion of the export of PV products from Vietnam. The third and fourth stages both contribute significantly, with 91.4% and 99.5%, respectively. Although its contribution rate was 62.03% in Phase V, its contribution was $2287.89 million, much larger than that of Phase IV.

In 2008–2009, the overall competitive effect had a damping effect on export growth of up to 89.55%, outperforming the specific competitive effect. It is clear that the overall competitiveness decline throughout this time period had a major negative impact on export growth. The quality of Vietnamese PV goods improved over this time, making them more competitive and progressively boosting export growth. However, from 2012 to 2017, the overall competitiveness quickly grew to 99.44%, and the specific competition effect was also beneficial.

#### ③ The contribution of second-order effects is small, and the export structure is not well adapted to the import demand of the world market

The second-order effect decreased from 63.59% in the first stage to 21.33% in the fifth stage, which shows that the second-order effect is contributing less and less to export growth. The pure second-order utility is positive in all five stages, implying that the change in the share of Vietnamese PV exports can adapt to the change in world import demand and positively pull export growth.

The dynamic second-order effect was positive in the first and second periods, contributing 5.54% and 1% respectively to export growth, the last three stages are all negative, implying that although competitiveness improved in these two periods, the export structure was not well adapted to world market import demand, and therefore the combined effect of the two affected export growth.

### (8) Chile

Exports showed an increase in the first four phases. The reason for the increase in exports in the second phase is that although the financial crisis had a dampening effect on the structure of Chilean solar photovoltaic exports, the competitiveness of Chilean solar photovoltaic products increased at this time, thus driving export growth. The fifth stage saw a sharp decrease in Chilean solar PV exports. This is mainly influenced by the new crown epidemic. The specific data of CMS model is shown in Table 9.

**Table 9:** Results of the CMS model decomposition of Chile’s solar PV export growth(US$ million).

Year	2001–2007		2008–2009		2010–2011		2012–2017		2018–2021	
	Amount of contribution	Contribution rate	Amount of contribution	Contribution rate	Amount of contribution	Contribution rate	Amount of contribution	Contribution rate	Amount of contribution	Contribution rate
Growth in export value	2.12	100.00%	0.41	100.00%	0.31	100.00%	7.74	100.00%	-1.89	100.00%
Structural effects	1.43	67.47%	-0.98	-235.58%	0.46	146.55%	0.05	0.69%	3.00	-158.44%
Product effects	-0.29	-13.75%	-0.28	-66.64%	0.2	62.61%	0.01	0.12%	-0.13	6.68%
Growth effects	1.73	81.22%	-0.7	-168.95%	0.26	83.94%	0.04	0.57%	3.12	-165.11%
Competitive effects	0.36	16.99%	1.68	405.92%	0.03	9.59%	7.7	99.51%	-3.42	180.94%
Overall competitive effect	0.15	7.16%	1.29	310.51%	0.05	15.56%	7.66	98.91%	-3.79	200.48%
Specific competitive effects	0.21	9.83%	0.4	95.41%	-0.02	-5.97%	0.05	0.60%	0.37	-19.54%
Second order effects	0.33	15.54%	-0.29	-70.33%	-0.17	-56.14%	-0.02	-0.20%	-1.47	77.49%
Pure second-order effects	0.59	27.59%	-0.23	-54.33%	0	0.31%	0.04	0.52%	-1.10	58.33%
Dynamic second-order effects	-0.26	-12.05%	-0.07	-16.00%	-0.18	-56.45%	-0.06	-0.72%	-0.36	19.17%

Source: Authors’ calculations based on UN comtrade database data using CMS model.

#### ① The role of rising world market demand for Chilean PV products in export growth is gradually becoming weaker

The structural effect, or the growth in global demand for solar photovoltaic products during this time period, was responsible for 64.74% of the increase in Chilean exports of photovoltaic products between 2001 and 2007. The structural impact of the financial crisis was negative in 2008–2009, adding 235.58% to export growth. At the same time, the global demand for PV products also decreased during this time, which had a negative impact on Chilean exports.

The growth effect negated the dampening effect of the product effect on export growth in the first phase, meaning that the main driver of export growth in this time period was global market demand, which Chilean exports of PV products did not adequately satisfy. In the third period, the structural effect increased, but it quickly fell back down, going from 146% in the third period to 0.69% in the fourth. The structural effect’s overall influence is fading.

#### ② Competitiveness is gradually becoming the primary driver of export growth, and the export structure is better aligned with changes in market import demand

The competitiveness effect’s contribution to export growth was 16.99% between 2001 and 2007, climbed significantly between 2008 and 2009, fell between 2010 and 2011, and then increased again between 2012 and 2017 to reach 99.51%. Hence, there has been an overall upward trend in Chile’s competitiveness of solar photovoltaic products from 2001 to 2017.

The overall competition impact continues to contribute as much as the competitiveness effect and does so better than the individual competition effect. In other words, overall competitiveness has steadily taken over as the main factor driving export growth, showing the willingness of most nations to prefer to import PV products from Chile. From 2012 to 2017, 98.91% of the overall competition effect was generated. The export structure is better suited to changes in import demand in global markets because of the distinctive competition effect, with the exception of the third phase.

#### ③ The second-order effect is fluctuating and ultimately still has a dampening effect on export growth

The second order effect had a contribution of 15.54% in 2001–2007, which means that this effect contributed positively to the growth of Chile’s export value. In 2008–2009, the second-order effect was -70.33%, significantly hindering export growth. A second-order effect of -77.49% was observed in the fifth period, lower than the -56.14% in the third period. The competitiveness of Chilean solar PV products decreased during this time, and the dynamic second-order effect also had a dampening effect on export growth, which fell by 56.45% between 2010 and 2011. The pure second-order effect was 27.59% in the first period and the dynamic second-order effect was 12.05%. The competitiveness of Chilean solar PV products declined less during the fourth period, and the structure of its exports was better adapted to the changes in import demand in international markets than it was in the third period, according to the growing dynamic second order effect and pure second order effect.

### (9) Mexico

Mexico’s exports fluctuated over the four phases, with an overall upward trend. The specific data of CMS model is shown in Table 10.

**Table 10:** Results of the CMS model decomposition of Mexico’s solar PV export growth(US$ million).

Year	2001–2007		2008–2009		2010–2011		2012–2017		2018–2021	
	Amount of contribution	Contribution rate	Amount of contribution	Contribution rate	Amount of contribution	Contribution rate	Amount of contribution	Contribution rate	Amount of contribution	Contribution rate
Growth in export value	-188.98	100.00%	-34.7	100.00%	352.59	100.00%	-215.54	100.00%	323.50	100.00%
Structural effects	1314.75	-695.70%	-146.42	421.98%	43.43	12.32%	8.27	-3.84%	491.61	151.96%
Product effects	-378.06	200.05%	-6.49	18.71%	0.28	0.08%	-0.46	0.21%	-22.62	-6.99%
Growth effects	1692.81	-895.75%	-139.93	403.26%	43.14	12.24%	8.73	-4.05%	514.23	158.96%
Competitive effects	-692.12	366.24%	115.53	-332.95%	299.39	84.91%	-220.15	102.14%	-119.84	-37.05%
Overall competitive effect	-717.08	379.45%	121.49	-350.13%	299.83	85.04%	-223.11	103.51%	-144.23	-44.58%
Specific competitive effects	24.96	-13.21%	-5.96	17.18%	-0.44	-0.13%	2.96	-1.37%	24.39	7.54%
Second order effects	-811.61	429.46%	-3.81	10.97%	9.78	2.77%	-3.66	1.70%	-48.26	-14.92%
Pure second-order effects	-1124.17	594.86%	-15.46	44.57%	9.61	2.72%	-1.14	0.53%	-38.63	-11.94%
Dynamic second-order effects	312.56	-165.39%	11.66	-33.60%	0.18	0.05%	-2.51	1.17%	-9.63	-2.98%

Source: Authors’ calculations based on UN comtrade database data using CMS model.

#### ① The contribution of structural effects has fluctuated considerably and is currently contributing very little to export growth

The structural effect was the main source of growth in Mexico’s exports of solar photovoltaic products in the first and fifth phase. 695.70% of the structural effect was recorded in 2001–2007, but a dampening effect of 421.98% was generated in 2008–2009, indicating that the fall in world import demand during this period caused a sharp fall in Mexican exports. With the recovery of the world economy after the financial crisis, the effect rose from the third period implying that Mexico’s main export markets were concentrated in countries with higher demand.

The growth effect was greater than the product effect, particularly in the first and second phases, where it was -895.75% and -403.26%, respectively. This suggests that during these two phases, the sharp decline in the demand for solar PV imports on the global market significantly hampered the growth of Mexican exports. The product effect’s contribution to export growth was incredibly small in all phases, acting as a countervailing force on export growth. This suggests that the international market’s preference for solar PV products has changed and that Mexican exports have failed to make the necessary improvements in a timely manner.

#### ② Overall downward trend in competitiveness, with export structure more in line with changes in import demand in international markets

The contribution of the competitiveness effect to export growth was -366.24% in the period 2001–2007, with a slight increase in the periods 2008–2009 and 2010–2011. However, it remains negative in the period 2018–2021. The decline in competitiveness has had a reverse pull effect on the growth of Mexico’s PV exports.

The contribution of the overall competition effect remains in the same direction as the competitiveness effect, and the contribution rate is better than that of the specific competition effect. The overall competitiveness effect reached 350.13% in the period 2008–2009, meaning that overall competitiveness gradually became the primary driver of export growth. As a result of the financial crisis during this period, demand for PV products in the world market fell, eventually offsetting the export growth from increased competitiveness. With the exception of the 2nd-3rd phase, the contribution of the specific competitiveness effect was positive, indicating that the export structure was able to fit relatively well with the changes in import demand in the international market.

#### ③ The contribution of second-order effects has increased

The second-order effect for the period 2001–2007 was -429.46%, a negative figure indicating that the combination of PV goods’ falling competitiveness and Mexico’s own exports’ inability to respond to changes in market demand hindered the growth of Mexican PV exports. Pure second-order utilities had a dampening effect of -594.86% on the growth of Mexican PV exports, showing that the shift in Mexican exports’ share was very poorly adapted to the shift in the demand for PV imports on the global market.

The pure second-order effect, which had a positive impact on export growth, was the main reason the second-order effect was positive in 2010–2011, however it was only positive at an absolute value of 2.77%. This shows that between the years 2010 and 2011, changes in the proportion of Mexican exports started to respond to shifts in global demand, and that competitiveness also improved, both of which together helped to boost the value of Mexican exports.

## 6. Research conclusions and policy recommendations

### 6.1 Research findings

Some scholars have concluded that trade protection will weaken the competitiveness of a country’s photovoltaic products [45], but the specific size of the impact and whether there are other factors have not been concluded. In this study, CMS and IRCA index methods were used to obtain the changing trend of PV product competitiveness in CPTPP countries and the main factors affecting export. Specific conclusions are as follows:

1. In terms of structural effects, the contribution of structural effects to the growth of solar PV exports in each CPTPP country has been declining in recent years. The drop in global market demand led to a large decrease in the export of photovoltaic products, especially in the wake of the new crown outbreak. In CPTPP nations like Japan, Chile, Vietnam, and Singapore, export growth is being fueled less and less by rising global demand. This implies that each nation’s export goods and market setup must change to meet global demand. However, in most countries in the fifth phase, the competitiveness effect of PV products has a dampening effect on export growth, showing that the rise in export costs during the epidemic period due to epidemic control policies and other influences has decreased competitiveness.
2. It can be concluded that the structure and share of solar PV exports of CPTPP countries in recent years can adapt to the changes in international market demand because the competitiveness effect of some CPTPP countries has contributed to the growth of solar PV exports in recent years has had an upward trend, especially the overall competitiveness effect. However, there are some nations whose IRCA results show that the competitiveness of their solar PV exports is declining, such as Malaysia, Mexico, and so on. At the same time, IRCA results show that the comparative advantage of the majority of countries is increasing, such as Vietnam, Chile, Canada, and so forth.
3. Second-order effects show a diminishing trend in recent years with respect to the contribution of solar photovoltaic product export growth, showing that the interplay of market structure and competitiveness of the CPTPP nations on solar photovoltaic product export growth is waning. But in Canada, the second-order effect has grown recently, going from a first-stage dampening effect to a fourth-stage boost of 3.78%. This shows that the rise of Canadian PV exports has been influenced by the interaction between the enhanced competitiveness of PV products and the steady adaptation of the structure of Canada’s own exports to changes in market demand.
4. Power supply items are the products in which most CPTPP nations have a strong comparative advantage, meaning that CPTPP countries have a strong export competitiveness in this product, according to an analysis of the different types of solar photovoltaic products. On the other hand, solar cells have a weaker comparative advantage or a constant comparative advantage. The export competitiveness of solar photovoltaic products from CPTPP countries is unequal, it might be said. Also, the IRCA indices of the majority of nations are below 1, demonstrating a limited comparative advantage for PV products in CPTPP nations. This could be because of the outdated nature of solar PV technology and the absence of government subsidy policies in CPTPP countries. Future related research should be devoted to examining the primary factors affecting the competitiveness of solar PV exports of each country, such as in-depth analysis based on technological innovation perspective or factor endowment perspective alone, in order to provide more clarity.

### 6.2 Policy insights

#### (1) Grasp the changes in the structure of world import demand and optimize the layout of product categories

The study’s first conclusion shows that the CPTPP countries should take advantage of shifts in global market demand and expand their markets by concentrating their export markets on nations with a higher demand for solar PV products. Produce more products that can be sold and increase the proportion of exports on the global market at the same time, boosting the structural impacts’ overall contribution. Given that power goods have a sizable comparative advantage, CPTPP countries should continue to work to boost exports of this commodity while simultaneously enhancing the quality of other products and raising the proportion of exports of these items in order to raise overall competitiveness.

#### (2) Reduce the production cost of solar photovoltaic products and improve the competitive advantage of exports

The calculations in this study show that importing power from China rather than Australia is more affordable for Singapore. Even though the CPTPP nations’ solar PV product competitiveness has improved recently, half of the countries are still losing market share. The CPTPP countries should create and sell goods at reduced costs wherever possible since doing so will improve competitiveness. China has applied in writing to join the CPTPP, and if it does, it will greatly enhance the CPTPP’s ability to boost the export competitiveness of solar PV.

#### (3) Improve product quality and form your own brand effect to improve your core competitiveness

The CPTPP countries will undoubtedly face intense competition as a result of the increased worldwide attention on the renewable energy sector. The CPTPP nations should concentrate on raising the technology content and added value in order to enhance the terms of trade for solar PV products and satisfy customer demand preferences in order to increase their core competitiveness. By modernizing the manufacturing process and enhancing the quality inspection process, they should also concentrate on raising the caliber of solar photovoltaic products in order to compete on quality.

## Supporting information

S1 Appendix (DOCX)

## References

[1] Qi W, Xiong H, Peng X. How to Promote International Competitiveness of China’s Renewable Energy Products?-Based on SNA Theory[J]. Polish Journal of Environmental Studies, 2021, 30(5). Available from: doi: 10.15244/pjoes/130635

[2] Zhao Z, Zhang SY, Hubbard B, et al. The emergence of the solar photovoltaic power industry in China[J]. Renewable and Sustainable Energy Reviews, 2013, 21: 229–236. Available from: doi: 10.1016/j.rser.2012.12.066

[3] Chaisse J. Renewables re-energized? The internationalization of green energy investment rules and disputes[J]. The Journal of World Energy Law & Business, 2016, 9(4): 269–281. Available from: doi: 10.1093/jwelb/jww018

[4] LE TAT. The Impact of Tariffs on Vietnam’s Trade in the Comprehensive and Progressive Agreement for Trans-Pacific Partnership (CPTPP)[J]. The Journal of Asian Finance, Economics and Business, 2021, 8(3): 771–780. Available from: doi: 10.13106/jafeb.2021.vol8.no3.0771

[5] Minh Thong L, Van Hiep T, Thi Thu Thuy B, et al. The competition possibility between renewable energy and fossil energy in Vietnam in the future[J]. The Journal of World Energy Law & Business, 2021, 14(3): 215–228. Available from: doi: 10.1093/jwelb/jwab021

[6] Su B, Goh T, Ang B W, et al. Energy consumption and energy efficiency trends in Singapore: The case of a meticulously planned city[J]. Energy Policy, 2022, 161: 112732. Available from: doi: 10.1016/j.enpol.2021.112732

[7] Vo DH, Tran Q, Tran T. Economic growth, renewable energy and financial development in the CPTPP countries[J]. Plos one, 2022, 17(6): e0268631. Available from: doi: 10.1371/journal.pone.0268631 35709183 PMC9202881

[8] Wang WF. Promoting green governance synergy of emerging regional trade agreements: A case study of RCEP and CPTPP[J]. International trade, 2022 (10): 34–42.

[9] Vo DH, Vo AT, Ho CM, et al. The role of renewable energy, alternative and nuclear energy in mitigating carbon emissions in the CPTPP countries[J]. Renewable Energy, 2020, 161: 278–292. Available from: doi: 10.1016/j.renene.2020.07.093

[10] Zhu XH, Ding Q, Chen JY. How does critical mineral trade pattern affect renewable energy development? The mediating role of renewable energy technological progress[J]. Energy Economics, 2022, 112. Available from: doi: 10.1016/j.eneco.2022.106164

[11] Fu JY, Wu LM. Do institutional and environmental policies affect the export trade of renewable energy industry? Based on the perspective of export depth and breadth[J]. Journal of International Trade,2015(12):85–95.

[12] Chowdhury MS, Rahman KS, Chowdhury T, et al. An overview of solar photovoltaic panels’ end-of-life material recycling[J]. Energy Strategy Reviews, 2020, 27: 100431.

[13] Shuai J, Zhao Y, Wang Y, et al. Renewable energy product competitiveness: Evidence from the United States, China and India[J]. Energy, 2022, 249: 123614. Available from: doi: 10.1016/j.energy.2022.123614

[14] Al-Shahri OA, Ismail FB, Hannan MA, et al. Solar photovoltaic energy optimization methods, challenges and issues: A comprehensive review[J]. Journal of Cleaner Production, 2021, 284: 125465. Available from: doi: 10.1016/j.jclepro.2020.125465

[15] Hossain M, Huda ASN, Mekhilef S, et al. A state-of-the-art review of hydropower in Malaysia as renewable energy: Current status and future prospects[J]. Energy strategy reviews, 2018, 22: 426–437. Available from: doi: 10.1016/j.esr.2018.11.001

[16] Lau HC, Zhang K, Bokka HK, et al. A review of the status of fossil and renewable energies in Southeast Asia and its implications on the decarbonization of ASEAN[J]. Energies, 2022, 15(6): 2152. Available from: doi: 10.3390/en15062152

[17] Daren G. Financial Challenges Impacting the Renewable Energy Industry[J]. Power Engineering, 2018(4).

[18] Gielen D, Boshell F, Saygin D, et al. The role of renewable energy in the global energy transformation[J]. Energy strategy reviews, 2019, 24: 38–50. Available from: doi: 10.1016/j.esr.2019.01.006

[19] Calise F, Cappiello FL, Cimmino L, et al. A review of the state of the art of biomethane production: recent advancements and integration of renewable energies[J]. Energies, 2021, 14(16): 4895. Available from: doi: 10.3390/en14164895

[20] Wang YL, Shao S. Research on the Fiscal and Taxation Policies to Promote the Development of Renewable Energy[C]//Advanced Materials Research. Trans Tech Publications Ltd, 2015, 1073: 2483–2487. Available from: doi: 10.4028/www.scientific.net/AMR.1073-1076.2483

[21] Boke MT, Moges SA, Dejen ZA. Optimizing renewable-based energy supply options for power generation in Ethiopia[J]. Plos one, 2022, 17(1): e0262595. Available from: doi: 10.1371/journal.pone.0262595 35030223 PMC8759650

[22] Mirletz H, Ovaitt S, Sridhar S, et al. Circular economy priorities for photovoltaics in the energy transition[J]. Plos one, 2022, 17(9): e0274351. Available from: doi: 10.1371/journal.pone.0274351 36083874 PMC9462576

[23] Zhao Y, Wang L, Yu Y. Trade liberalization and China’s exports of renewable energy products: Evidence from product level data[J]. Emerging Markets Finance and Trade, 2016, 52(6): 1281–1297. Available from: doi: 10.1080/1540496X.2016.1152788

[24] Chang PL, Nguyen PTB. Global value chains and the CPTPP[J]. The World Economy, 2022. Available from: doi: 10.1111/twec.13300

[25] Qian X. Phoenix from the Ashes: CPTPP Meaning for Asia-Pacific (and Global) Investment[J]. Asian J. WTO & Int’l Health L & Pol’y, 2020, 15: 567.

[26] Jung J. The Effects of China’s Participation in the Comprehensive and Progressive Agreement for Trans-Pacific Partnership (CPTPP): A Quantitative Assessment[J]. Sustainability, 2022, 15(1): 344. Available from: doi: 10.3390/su15010344

[27] Marcoux J M, Sylvestre-Fleury J. China’s contestation of international norms on state-owned enterprises and government procurement through the Belt and Road Initiative[J]. Asia Pacific Law Review, 2022, 30(2): 325–347. Available from: doi: 10.1080/10192557.2022.2085413

[28] Chaisse J, Gao H, Lo C. Paradigm Shift in International Economic Law Rule-Making[M]. Springer, 2017.

[29] Sun J. Study on international trade competitiveness of solar photovoltaic industry[J]. Price Monthly, 2017(12):32–36.

[30] Wang M, Mao X, Xing Y, et al. Breaking down barriers on PV trade will facilitate global carbon mitigation[J]. Nature communications, 2021, 12(1):1–16. 10.1038/s41467-021-26547-7 PMC8613243 34819496

[31] Shuai J, Chen C, Cheng J, et al. Are China’s solar PV products competitive in the context of the Belt and Road Initiative?[J]. Energy policy, 2018, 120: 559–568. Available from: doi: 10.1016/j.enpol.2018.05.042

[32] Zhao ZY, Yang HJ, Zuo J. Evolution of international trade for photovoltaic cells: A spatial structure study[J]. Energy, 2017, 124: 435–446. Available from: doi: 10.1016/j.energy.2017.02.093

[33] Li XD, Wu YW. Re-measurement of China’s manufacturing industry trade competitiveness by industry—a comparison based on RCA index and NRCA index[J]. Research World, 2021(01): 39–47.

[34] Mansourzadeh MJ, Shahmoradi B, Dehdarirad H, et al. A note on using revealed comparative advantages in scientometrics studies[J]. Scientometrics, 2019, 121(1): 595–599. Available from: doi: 10.1007/s11192-019-03207-8

[35] Wu HW, Zhang SX, Liu MY. A study on trade competitiveness between China and ASEAN from the perspective of "One Belt and One Road": Based on the improved Explicit Comparative Advantage Index[J]. International Economic Cooperation, 2019(06): 53–61.

[36] Zhang B, Yang LA, Yang FL, Wang H, Xie XJ, et al. Based on projection pursuit of the comprehensive evaluation of soil nutrients and influencing factors of study[J]. Journal of soil, 2020, 52 (6): 1239–1247.

[37] Liu YY, Tao CQ. Calculation and analysis of the development level of digital trade in 31 provinces of China: Based on RAGA projection pursuit model[J]. The price issue, 2021 (4): 69–76.

[38] Zhang ZQ, Song X, Fu JX. Urban road cross-section evaluation method based on projection tracing method[J]. Journal of Chongqing Jiaotong University (Natural Science Edition), 2022, 41(12): 41–47+93.

[39] Liu XY, Li MF. CMS model analysis on Influencing factors of apple export in China[J]. China Fruit Trees, 2022(12): 99–103.

[40] Qin JN, Zhang JB, Zhao DJ, Wu YF. Analysis of Influencing Factors of Chinese edible fungi Export to RCEP countries based on CMS model[J]. Journal of Edible Fungi, 2002, 29(05): 109–122.

[41] Zhang Y, Zhang WL. Causes of sunflower product trade changes in China: An empirical analysis based on CMS model[J]. World Agriculture, 2020(07): 53–60+84.

[42] Zhe GC, Han LX. Analysis on the Fluctuation Factors of China’s Ceramic Export Trade: Based on the analysis perspective of CMS Model[J]. International Economic and Trade Exploration, 2016, 32(09): 40–57.

[43] Ge M, GAO YD, Zhao S P. Research on Driving Factors of China’s agricultural Export growth under the framework of RCEP: Based on CMS three-level decomposition model[J/OL]. Agricultural Technical economics:1–17[2023-01-31].

[44] Liu SY, Li DH. Analysis on the Causes of the fluctuation of tea export trade between China and the United States: Based on modified CMS Model[J]. Science of Tea, 2021, 41(06): 876–888.

[45] Chen SQ, Liu XD. International comparison of photovoltaic industry competitiveness: An empirical analysis based on panel data[J]. Scientific Decision, 2016(01): 77–94.

