# Supplementary material for: Assessing the competitiveness of solar photovoltaic products in comprehensive and progressive agreement for trans-pacific partnership countries
Source: PLoS One. 2023 Jul 27;18(7):e0284783. doi: 10.1371/journal.pone.0284783 (PMC10374145; doi:10.1371/journal.pone.0284783)
Supplement: S1 Appendix — (DOCX) [file pone.0284783.s001.docx]

Australian trade data

| Code | Year | Total exports of the product from the country | Total exports of the country | Total world exports of the product | Total world export |
| --- | --- | --- | --- | --- | --- |
| HS850440 | 2001 | 6969207 | 63288188610 | 15277808966 | 6.06563E+12 |
|  | 2002 | 14388620 | 64984167265 | 14331199364 | 6.38013E+12 |
|  | 2003 | 17081678 | 70288235404 | 16470599661 | 7.45788E+12 |
|  | 2004 | 16608152 | 86484037752 | 19943301119 | 9.02332E+12 |
|  | 2005 | 15242767 | 1.06011E+11 | 21905426253 | 1.01628E+13 |
|  | 2006 | 19277444 | 1.23324E+11 | 26743646603 | 1.18798E+13 |
|  | 2007 | 31585432 | 1.41182E+11 | 33152966500 | 1.36111E+13 |
|  | 2008 | 49746539 | 1.86853E+11 | 37779806328 | 1.56856E+13 |
|  | 2009 | 36380580 | 1.539E+11 | 32380692622 | 1.22547E+13 |
|  | 2010 | 35672823 | 2.12109E+11 | 44068548896 | 1.49017E+13 |
|  | 2011 | 49067443 | 2.69423E+11 | 49441655896 | 1.78992E+13 |
|  | 2012 | 50629144 | 2.56243E+11 | 49668324792 | 1.7838E+13 |
|  | 2013 | 51777965 | 2.52155E+11 | 52352958739 | 1.85519E+13 |
|  | 2014 | 54947947 | 2.40445E+11 | 53327179708 | 1.846E+13 |
|  | 2015 | 55078401 | 1.87792E+11 | 51252405508 | 1.61321E+13 |
|  | 2016 | 60750830 | 1.8963E+11 | 48946091849 | 1.56794E+13 |
|  | 2017 | 76584311 | 2.30537E+11 | 52930978543 | 1.7271E+13 |
|  | 2018 | 94620393 | 2.52758E+11 | 58249024481 | 1.89541E+13 |
|  | 2019 | 88452738 | 2.66377E+11 | 60141078135 | 1.836E+13 |
|  | 2020 | 94636928 | 2.45046E+11 | 64964866446 | 1.71299E+13 |
|  | 2021 | 127744095 | 3.42036E+11 | 82970404994 | 2.15086E+13 |
| HS854140 | 2001 | 9578279 | 63288188610 | 6713998933 | 6.06563E+12 |
|  | 2002 | 23637458 | 64984167265 | 7179332265 | 6.38013E+12 |
|  | 2003 | 44862642 | 70288235404 | 9932685509 | 7.45788E+12 |
|  | 2004 | 64604278 | 86484037752 | 13005465288 | 9.02332E+12 |
|  | 2005 | 67424919 | 1.06011E+11 | 15762738743 | 1.01628E+13 |
|  | 2006 | 85131341 | 1.23324E+11 | 20416084673 | 1.18798E+13 |
|  | 2007 | 104106902 | 1.41182E+11 | 27328567002 | 1.36111E+13 |
|  | 2008 | 112585009 | 1.86853E+11 | 44075301249 | 1.56856E+13 |
|  | 2009 | 29634372 | 1.539E+11 | 39324527570 | 1.22547E+13 |
|  | 2010 | 16563952 | 2.12109E+11 | 72406848962 | 1.49017E+13 |
|  | 2011 | 58587540 | 2.69423E+11 | 74168174379 | 1.78992E+13 |
|  | 2012 | 18989654 | 2.56243E+11 | 53816544857 | 1.7838E+13 |
|  | 2013 | 22268827 | 2.52155E+11 | 50295528204 | 1.85519E+13 |
|  | 2014 | 19317198 | 2.40445E+11 | 54280798128 | 1.846E+13 |
|  | 2015 | 22486658 | 1.87792E+11 | 56868728529 | 1.61321E+13 |
|  | 2016 | 17462886 | 1.8963E+11 | 53121579803 | 1.56794E+13 |
|  | 2017 | 17115608 | 2.30537E+11 | 52130102560 | 1.7271E+13 |
|  | 2018 | 17781547 | 2.52758E+11 | 58249024481 | 1.89541E+13 |
|  | 2019 | 17971909 | 2.66377E+11 | 60141078135 | 1.836E+13 |
|  | 2020 | 14477203 | 2.45046E+11 | 64964866446 | 1.71299E+13 |
|  | 2021 | 19760113 | 3.42036E+11 | 82970404994 | 2.15086E+13 |

Canadian trade data

| Code | Year | Total exports of the product from the country | Total exports of the country | Total world exports of the product | Total world export |
| --- | --- | --- | --- | --- | --- |
| HS850440 | 2001 | 298208184 | 2.61059E+11 | 15277808966 | 6.06563E+12 |
|  | 2002 | 293213118 | 2.52584E+11 | 14331199364 | 6.38013E+12 |
|  | 2003 | 277094111 | 2.7223E+11 | 16470599661 | 7.45788E+12 |
|  | 2004 | 341483237 | 3.17161E+11 | 19943301119 | 9.02332E+12 |
|  | 2005 | 372809388 | 3.60552E+11 | 21905426253 | 1.01628E+13 |
|  | 2006 | 433362030 | 3.88179E+11 | 26743646603 | 1.18798E+13 |
|  | 2007 | 455654496 | 4.19882E+11 | 33152966500 | 1.36111E+13 |
|  | 2008 | 474205847 | 4.55632E+11 | 37779806328 | 1.56856E+13 |
|  | 2009 | 382998499 | 3.15177E+11 | 32380692622 | 1.22547E+13 |
|  | 2010 | 472222447 | 3.8658E+11 | 44068548896 | 1.49017E+13 |
|  | 2011 | 509351251 | 4.5043E+11 | 49441655896 | 1.78992E+13 |
|  | 2012 | 489423715 | 4.54099E+11 | 49668324792 | 1.7838E+13 |
|  | 2013 | 470723759 | 4.56598E+11 | 52352958739 | 1.85519E+13 |
|  | 2014 | 445711034 | 4.75177E+11 | 53327179708 | 1.846E+13 |
|  | 2015 | 385158315 | 4.08697E+11 | 51252405508 | 1.61321E+13 |
|  | 2016 | 382101966 | 3.88853E+11 | 48946091849 | 1.56794E+13 |
|  | 2017 | 414518041 | 4.20074E+11 | 52930978543 | 1.7271E+13 |
|  | 2018 | 444396552 | 4.50392E+11 | 58249024481 | 1.89541E+13 |
|  | 2019 | 412606632 | 4.45493E+11 | 60141078135 | 1.836E+13 |
|  | 2020 | 319904627 | 3.88377E+11 | 64964866446 | 1.71299E+13 |
|  | 2021 | 344819466 | 5.01201E+11 | 82970404994 | 2.15086E+13 |
| HS854140 | 2001 | 61907337 | 2.61059E+11 | 6713998933 | 6.06563E+12 |
|  | 2002 | 55696747 | 2.52584E+11 | 7179332265 | 6.38013E+12 |
|  | 2003 | 79572958 | 2.7223E+11 | 9932685509 | 7.45788E+12 |
|  | 2004 | 56658186 | 3.17161E+11 | 13005465288 | 9.02332E+12 |
|  | 2005 | 62063978 | 3.60552E+11 | 15762738743 | 1.01628E+13 |
|  | 2006 | 74555571 | 3.88179E+11 | 20416084673 | 1.18798E+13 |
|  | 2007 | 93165336 | 4.19882E+11 | 27328567002 | 1.36111E+13 |
|  | 2008 | 162737349 | 4.55632E+11 | 44075301249 | 1.56856E+13 |
|  | 2009 | 86364987 | 3.15177E+11 | 39324527570 | 1.22547E+13 |
|  | 2010 | 93918132 | 3.8658E+11 | 72406848962 | 1.49017E+13 |
|  | 2011 | 105812330 | 4.5043E+11 | 74168174379 | 1.78992E+13 |
|  | 2012 | 162752394 | 4.54099E+11 | 53816544857 | 1.7838E+13 |
|  | 2013 | 113965544 | 4.56598E+11 | 50295528204 | 1.85519E+13 |
|  | 2014 | 155235048 | 4.75177E+11 | 54280798128 | 1.846E+13 |
|  | 2015 | 320461062 | 4.08697E+11 | 56868728529 | 1.61321E+13 |
|  | 2016 | 286684988 | 3.88853E+11 | 53121579803 | 1.56794E+13 |
|  | 2017 | 248941180 | 4.20074E+11 | 52130102560 | 1.7271E+13 |
|  | 2018 | 186390878 | 4.50392E+11 | 58249024481 | 1.89541E+13 |
|  | 2019 | 169612987 | 4.45493E+11 | 60141078135 | 1.836E+13 |
|  | 2020 | 229629367 | 3.88377E+11 | 64964866446 | 1.71299E+13 |
|  | 2021 | 206881631 | 5.01201E+11 | 82970404994 | 2.15086E+13 |

Trade data for Malaysia

| Code | Year | Total exports of the product from the country | Total exports of the country | Total world exports of the product | Total world export |
| --- | --- | --- | --- | --- | --- |
| HS850440 | 2001 | 250233513 | 88004108096 | 15277808966 | 6.07E+12 |
|  | 2002 | 156389345 | 93282453146 | 14331199364 | 6.38E+12 |
|  | 2003 | 166936439 | 1.05E+11 | 16470599661 | 7.46E+12 |
|  | 2004 | 219271606 | 1.27E+11 | 19943301119 | 9.02E+12 |
|  | 2005 | 204123948 | 1.42E+11 | 21905426253 | 1.02E+13 |
|  | 2006 | 247824694 | 1.61E+11 | 26743646603 | 1.19E+13 |
|  | 2007 | 266331495 | 1.76E+11 | 33152966500 | 1.36E+13 |
|  | 2008 | 238722533 | 1.99E+11 | 37779806328 | 1.57E+13 |
|  | 2009 | 177973065 | 1.57E+11 | 32380692622 | 1.23E+13 |
|  | 2010 | 235417486 | 1.99E+11 | 44068548896 | 1.49E+13 |
|  | 2011 | 258831627 | 2.27E+11 | 49441655896 | 1.79E+13 |
|  | 2012 | 477523243 | 2.27E+11 | 49668324792 | 1.78E+13 |
|  | 2013 | 491727164 | 2.28E+11 | 52352958739 | 1.86E+13 |
|  | 2014 | 432639361 | 2.34E+11 | 53327179708 | 1.85E+13 |
|  | 2015 | 356009053 | 2.00E+11 | 51252405508 | 1.61E+13 |
|  | 2016 | 341657303 | 1.89E+11 | 48946091849 | 1.57E+13 |
|  | 2017 | 384929332 | 2.18E+11 | 52930978543 | 1.73E+13 |
|  | 2018 | 494209864 | 2.48712E+11 | 58249024481 | 1.89541E+13 |
|  | 2019 | 603198542 | 2.40212E+11 | 60141078135 | 1.836E+13 |
|  | 2020 | 776113903 | 2.3405E+11 | 64964866446 | 1.71299E+13 |
|  | 2021 | 880743024 | 2.9923E+11 | 82970404994 | 2.15086E+13 |
| HS854140 | 2001 | 687790965 | 88004108096 | 6713998933 | 6.06563E+12 |
|  | 2002 | 622588712 | 93282453146 | 7179332265 | 6.38013E+12 |
|  | 2003 | 664010363 | 1.04969E+11 | 9932685509 | 7.45788E+12 |
|  | 2004 | 792746564 | 1.2664E+11 | 13005465288 | 9.02332E+12 |
|  | 2005 | 843817484 | 1.41624E+11 | 15762738743 | 1.01628E+13 |
|  | 2006 | 1004274133 | 1.60669E+11 | 20416084673 | 1.18798E+13 |
|  | 2007 | 942389380 | 1.75962E+11 | 27328567002 | 1.36111E+13 |
|  | 2008 | 744605248 | 1.98702E+11 | 44075301249 | 1.56856E+13 |
|  | 2009 | 835520924 | 1.57195E+11 | 39324527570 | 1.22547E+13 |
|  | 2010 | 2598663689 | 1.98791E+11 | 72406848962 | 1.49017E+13 |
|  | 2011 | 2725637481 | 2.26993E+11 | 74168174379 | 1.78992E+13 |
|  | 2012 | 2520040745 | 2.27449E+11 | 53816544857 | 1.7838E+13 |
|  | 2013 | 3288038832 | 2.28316E+11 | 50295528204 | 1.85519E+13 |
|  | 2014 | 3420862650 | 2.34135E+11 | 54280798128 | 1.846E+13 |
|  | 2015 | 3931029200 | 2.00211E+11 | 56868728529 | 1.61321E+13 |
|  | 2016 | 4386716151 | 1.89414E+11 | 53121579803 | 1.56794E+13 |
|  | 2017 | 4046084935 | 2.17723E+11 | 52130102560 | 1.7271E+13 |
|  | 2018 | 4512717856 | 2.48712E+11 | 58249024481 | 1.89541E+13 |
|  | 2019 | 4679690016 | 2.40212E+11 | 60141078135 | 1.836E+13 |
|  | 2020 | 4186494969 | 2.3405E+11 | 64964866446 | 1.71299E+13 |
|  | 2021 | 4883870902 | 2.9923E+11 | 82970404994 | 2.15086E+13 |
| HS841990 | 2001 | 14112393 | 88004108096 | 2443086166 | 6.06563E+12 |
|  | 2002 | 8704810 | 93282453146 | 2412170325 | 6.38013E+12 |
|  | 2003 | 6558913 | 1.04969E+11 | 2825555174 | 7.45788E+12 |
|  | 2004 | 13899852 | 1.2664E+11 | 3455050913 | 9.02332E+12 |
|  | 2005 | 26569658 | 1.41624E+11 | 4149219879 | 1.01628E+13 |
|  | 2006 | 9108212 | 1.60669E+11 | 4963291717 | 1.18798E+13 |
|  | 2007 | 18585398 | 1.75962E+11 | 6841039110 | 1.36111E+13 |
|  | 2008 | 25150248 | 1.98702E+11 | 7067302136 | 1.56856E+13 |
|  | 2009 | 46525634 | 1.57195E+11 | 5609714551 | 1.22547E+13 |
|  | 2010 | 18458109 | 1.98791E+11 | 5523756597 | 1.49017E+13 |
|  | 2011 | 29554898 | 2.26993E+11 | 6414565689 | 1.78992E+13 |
|  | 2012 | 51528225 | 2.27449E+11 | 6720957298 | 1.7838E+13 |
|  | 2013 | 63959380 | 2.28316E+11 | 6816610839 | 1.85519E+13 |
|  | 2014 | 72591067 | 2.34135E+11 | 6759789698 | 1.846E+13 |
|  | 2015 | 77547640 | 2.00211E+11 | 6282411116 | 1.61321E+13 |
|  | 2016 | 84494606 | 1.89414E+11 | 6147822578 | 1.56794E+13 |
|  | 2017 | 93396110 | 2.17723E+11 | 6138200431 | 1.7271E+13 |
|  | 2018 | 56934074 | 2.48712E+11 | 58249024481 | 1.89541E+13 |
|  | 2019 | 65832227 | 2.40212E+11 | 60141078135 | 1.836E+13 |
|  | 2020 | 67352665 | 2.3405E+11 | 64964866446 | 1.71299E+13 |
|  | 2021 | 118467391 | 2.9923E+11 | 82970404994 | 2.15086E+13 |

Trade data for Peru

| Code | Year | Total exports of the product from the country | Total exports of the country | Total world exports of the product | Total world export |
| --- | --- | --- | --- | --- | --- |
| HS850440 | 2001 | 133794 | 6825610607 | 15277808966 | 6.06563E+12 |
|  | 2002 | 310110 | 7665213522 | 14331199364 | 6.38013E+12 |
|  | 2003 | 328276 | 9026639026 | 16470599661 | 7.45788E+12 |
|  | 2004 | 1006946 | 12726496792 | 19943301119 | 9.02332E+12 |
|  | 2005 | 3384935 | 17114288769 | 21905426253 | 1.01628E+13 |
|  | 2006 | 457089 | 23764896761 | 26743646603 | 1.18798E+13 |
|  | 2007 | 525072 | 28084585255 | 33152966500 | 1.36111E+13 |
|  | 2008 | 444110 | 31288211596 | 37779806328 | 1.56856E+13 |
|  | 2009 | 1314368 | 26738259539 | 32380692622 | 1.22547E+13 |
|  | 2010 | 1180845 | 35807438494 | 44068548896 | 1.49017E+13 |
|  | 2011 | 852969 | 46386021919 | 49441655896 | 1.78992E+13 |
|  | 2012 | 1186839 | 46366535800 | 49668324792 | 1.7838E+13 |
|  | 2013 | 1454280 | 42568898535 | 52352958739 | 1.85519E+13 |
|  | 2014 | 1405823 | 38645855012 | 53327179708 | 1.846E+13 |
|  | 2015 | 1057627 | 33667006869 | 51252405508 | 1.61321E+13 |
|  | 2016 | 1070821 | 36309958803 | 48946091849 | 1.56794E+13 |
|  | 2017 | 1795189 | 44237949422 | 52930978543 | 1.7271E+13 |
|  | 2018 | 1381641 | 48015148375 | 58249024481 | 1.89541E+13 |
|  | 2019 | 1395726 | 46131564759 | 60141078135 | 1.836E+13 |
|  | 2020 | 1284515 | 38757234044 | 64964866446 | 1.71299E+13 |
|  | 2021 | 934718 | 56260115202 | 82970404994 | 2.15086E+13 |
| HS854140 | 2001 | 5006 | 6825610607 | 6713998933 | 6.06563E+12 |
|  | 2002 | 3879 | 7665213522 | 7179332265 | 6.38013E+12 |
|  | 2003 | 131979 | 9026639026 | 9932685509 | 7.45788E+12 |
|  | 2004 | 18854 | 12726496792 | 13005465288 | 9.02332E+12 |
|  | 2005 | 8419 | 17114288769 | 15762738743 | 1.01628E+13 |
|  | 2006 | 108549 | 23764896761 | 20416084673 | 1.18798E+13 |
|  | 2007 | 61804 | 28084585255 | 27328567002 | 1.36111E+13 |
|  | 2008 | 42724 | 31288211596 | 44075301249 | 1.56856E+13 |
|  | 2009 | 235829 | 26738259539 | 39324527570 | 1.22547E+13 |
|  | 2010 | 6239 | 35807438494 | 72406848962 | 1.49017E+13 |
|  | 2011 | 26072 | 46386021919 | 74168174379 | 1.78992E+13 |
|  | 2012 | 3442 | 46366535800 | 53816544857 | 1.7838E+13 |
|  | 2013 | 8100 | 42568898535 | 50295528204 | 1.85519E+13 |
|  | 2014 | 300972 | 38645855012 | 54280798128 | 1.846E+13 |
|  | 2015 | 32749 | 33667006869 | 56868728529 | 1.61321E+13 |
|  | 2016 | 29241 | 36309958803 | 53121579803 | 1.56794E+13 |
|  | 2017 | 45376 | 44237949422 | 52130102560 | 1.7271E+13 |
|  | 2018 | 32749 | 48015148375 | 52022745429 | 1.89541E+13 |
|  | 2019 | 29241 | 46131564759 | 56432968212 | 1.836E+13 |
|  | 2020 | 45376 | 38757234044 | 58322340287 | 1.71299E+13 |
|  | 2021 | 100828 | 56260115202 | 73878931267 | 2.15086E+13 |
| HS841990 | 2001 | 2872 | 6825610607 | 2443086166 | 6.06563E+12 |
|  | 2002 | 45971 | 7665213522 | 2412170325 | 6.38013E+12 |
|  | 2003 | 205701 | 9026639026 | 2825555174 | 7.45788E+12 |
|  | 2004 | 266258 | 12726496792 | 3455050913 | 9.02332E+12 |
|  | 2005 | 518470 | 17114288769 | 4149219879 | 1.01628E+13 |
|  | 2006 | 502985 | 23764896761 | 4963291717 | 1.18798E+13 |
|  | 2007 | 912485 | 28084585255 | 6841039110 | 1.36111E+13 |
|  | 2008 | 1184445 | 31288211596 | 7067302136 | 1.56856E+13 |
|  | 2009 | 1230898 | 26738259539 | 5609714551 | 1.22547E+13 |
|  | 2010 | 808955 | 35807438494 | 5523756597 | 1.49017E+13 |
|  | 2011 | 1039083 | 46386021919 | 6414565689 | 1.78992E+13 |
|  | 2012 | 581518 | 46366535800 | 6720957298 | 1.7838E+13 |
|  | 2013 | 2015919 | 42568898535 | 6816610839 | 1.85519E+13 |
|  | 2014 | 989968 | 38645855012 | 6759789698 | 1.846E+13 |
|  | 2015 | 846766 | 33667006869 | 6282411116 | 1.61321E+13 |
|  | 2016 | 1340976 | 36309958803 | 6147822578 | 1.56794E+13 |
|  | 2017 | 1323499 | 44237949422 | 6138200431 | 1.7271E+13 |
|  | 2018 | 1539042 | 48015148375 | 6367541909 | 1.89541E+13 |
|  | 2019 | 1559068 | 46131564759 | 6388526721 | 1.836E+13 |
|  | 2020 | 1236742 | 38757234044 | 5938406474 | 1.71299E+13 |
|  | 2021 | 877655 | 56260115202 | 6724014953 | 2.15086E+13 |

Trade data from Mexico

| Code | Year | Total exports of the product from the country | Total exports of the country | Total world exports of the product | Total world export |
| --- | --- | --- | --- | --- | --- |
| HS850440 | 2001 | 966573305 | 1.58386E+11 | 15277808966 | 6.06563E+12 |
|  | 2002 | 805828720 | 1.60751E+11 | 14331199364 | 6.38013E+12 |
|  | 2003 | 768784801 | 1.64907E+11 | 16470599661 | 7.45788E+12 |
|  | 2004 | 687678790 | 1.8798E+11 | 19943301119 | 9.02332E+12 |
|  | 2005 | 639315853 | 2.14207E+11 | 21905426253 | 1.01628E+13 |
|  | 2006 | 691577002 | 2.49961E+11 | 26743646603 | 1.18798E+13 |
|  | 2007 | 578365063 | 2.71821E+11 | 33152966500 | 1.36111E+13 |
|  | 2008 | 573307908 | 2.91265E+11 | 37779806328 | 1.56856E+13 |
|  | 2009 | 394036118 | 2.29712E+11 | 32380692622 | 1.22547E+13 |
|  | 2010 | 581989305 | 2.98305E+11 | 44068548896 | 1.49017E+13 |
|  | 2011 | 694353912 | 3.49327E+11 | 49441655896 | 1.78992E+13 |
|  | 2012 | 845717143 | 3.70707E+11 | 49668324792 | 1.7838E+13 |
|  | 2013 | 922372696 | 3.79949E+11 | 52352958739 | 1.85519E+13 |
|  | 2014 | 1000082312 | 3.9689E+11 | 53327179708 | 1.846E+13 |
|  | 2015 | 1072046764 | 3.80556E+11 | 51252405508 | 1.61321E+13 |
|  | 2016 | 1181662458 | 3.73954E+11 | 48946091849 | 1.56794E+13 |
|  | 2017 | 1249645530 | 4.09396E+11 | 52930978543 | 1.7271E+13 |
|  | 2018 | 1366950438 | 4.50684E+11 | 58249024481 | 1.89541E+13 |
|  | 2019 | 1528450665 | 4.60604E+11 | 60141078135 | 1.836E+13 |
|  | 2020 | 1528550384 | 4.16982E+11 | 64964866446 | 1.71299E+13 |
|  | 2021 | 1726037457 | 4.94596E+11 | 82970404994 | 2.15086E+13 |
| HS854140 | 2001 | 58205984 | 1.58386E+11 | 6713998933 | 6.06563E+12 |
|  | 2002 | 46698730 | 1.60751E+11 | 7179332265 | 6.38013E+12 |
|  | 2003 | 64292328 | 1.64907E+11 | 9932685509 | 7.45788E+12 |
|  | 2004 | 81645502 | 1.8798E+11 | 13005465288 | 9.02332E+12 |
|  | 2005 | 140839172 | 2.14207E+11 | 15762738743 | 1.01628E+13 |
|  | 2006 | 218546017 | 2.49961E+11 | 20416084673 | 1.18798E+13 |
|  | 2007 | 200562240 | 2.71821E+11 | 27328567002 | 1.36111E+13 |
|  | 2008 | 397613493 | 2.91265E+11 | 44075301249 | 1.56856E+13 |
|  | 2009 | 560096909 | 2.29712E+11 | 39324527570 | 1.22547E+13 |
|  | 2010 | 710974039 | 2.98305E+11 | 72406848962 | 1.49017E+13 |
|  | 2011 | 931913503 | 3.49327E+11 | 74168174379 | 1.78992E+13 |
|  | 2012 | 751241218 | 3.70707E+11 | 53816544857 | 1.7838E+13 |
|  | 2013 | 775606300 | 3.79949E+11 | 50295528204 | 1.85519E+13 |
|  | 2014 | 616308950 | 3.9689E+11 | 54280798128 | 1.846E+13 |
|  | 2015 | 921145572 | 3.80556E+11 | 56868728529 | 1.61321E+13 |
|  | 2016 | 883383851 | 3.73954E+11 | 53121579803 | 1.56794E+13 |
|  | 2017 | 139445973 | 4.09396E+11 | 52130102560 | 1.7271E+13 |
|  | 2018 | 130098592 | 4.50684E+11 | 58249024481 | 1.89541E+13 |
|  | 2019 | 129766457 | 4.60604E+11 | 60141078135 | 1.836E+13 |
|  | 2020 | 98834277 | 4.16982E+11 | 64964866446 | 1.71299E+13 |
|  | 2021 | 79688165 | 4.94596E+11 | 82970404994 | 2.15086E+13 |
| HS841990 | 2001 | 17440679 | 1.58386E+11 | 2443086166 | 6.06563E+12 |
|  | 2002 | 24140961 | 1.60751E+11 | 2412170325 | 6.38013E+12 |
|  | 2003 | 27050543 | 1.64907E+11 | 2825555174 | 7.45788E+12 |
|  | 2004 | 27294871 | 1.8798E+11 | 3455050913 | 9.02332E+12 |
|  | 2005 | 44525836 | 2.14207E+11 | 4149219879 | 1.01628E+13 |
|  | 2006 | 53845461 | 2.49961E+11 | 4963291717 | 1.18798E+13 |
|  | 2007 | 74311391 | 2.71821E+11 | 6841039110 | 1.36111E+13 |
|  | 2008 | 74475626 | 2.91265E+11 | 7067302136 | 1.56856E+13 |
|  | 2009 | 56564422 | 2.29712E+11 | 5609714551 | 1.22547E+13 |
|  | 2010 | 51615196 | 2.98305E+11 | 5523756597 | 1.49017E+13 |
|  | 2011 | 70905793 | 3.49327E+11 | 6414565689 | 1.78992E+13 |
|  | 2012 | 82570216 | 3.70707E+11 | 6720957298 | 1.7838E+13 |
|  | 2013 | 76630321 | 3.79949E+11 | 6816610839 | 1.85519E+13 |
|  | 2014 | 96075938 | 3.9689E+11 | 6759789698 | 1.846E+13 |
|  | 2015 | 79941438 | 3.80556E+11 | 6282411116 | 1.61321E+13 |
|  | 2016 | 61893856 | 3.73954E+11 | 6147822578 | 1.56794E+13 |
|  | 2017 | 74896211 | 4.09396E+11 | 6138200431 | 1.7271E+13 |
|  | 2018 | 98185296 | 4.50684E+11 | 58249024481 | 1.89541E+13 |
|  | 2019 | 84004430 | 4.60604E+11 | 60141078135 | 1.836E+13 |
|  | 2020 | 71611537 | 4.16982E+11 | 64964866446 | 1.71299E+13 |
|  | 2021 | 113013616 | 4.94596E+11 | 82970404994 | 2.15086E+13 |

Trade data from Japan

| Code | Year | Total exports of the product from the country | Total exports of the country | Total world exports of the product | Total world export |
| --- | --- | --- | --- | --- | --- |
| HS850440 | 2001 | 819573074 | 4.03344E+11 | 15277808966 | 6.06563E+12 |
|  | 2002 | 768977609 | 4.16729E+11 | 14331199364 | 6.38013E+12 |
|  | 2003 | 865046744 | 4.72007E+11 | 16470599661 | 7.45788E+12 |
|  | 2004 | 1035022904 | 5.65761E+11 | 19943301119 | 9.02332E+12 |
|  | 2005 | 1023364547 | 5.94941E+11 | 21905426253 | 1.01628E+13 |
|  | 2006 | 1178611460 | 6.46725E+11 | 26743646603 | 1.18798E+13 |
|  | 2007 | 1261427453 | 7.14327E+11 | 33152966500 | 1.36111E+13 |
|  | 2008 | 1462219117 | 7.81412E+11 | 37779806328 | 1.56856E+13 |
|  | 2009 | 1119856106 | 5.80719E+11 | 32380692622 | 1.22547E+13 |
|  | 2010 | 1563882836 | 7.69774E+11 | 44068548896 | 1.49017E+13 |
|  | 2011 | 2036734062 | 8.23184E+11 | 49441655896 | 1.78992E+13 |
|  | 2012 | 1906969370 | 7.9862E+11 | 49668324792 | 1.7838E+13 |
|  | 2013 | 1973767029 | 7.15097E+11 | 52352958739 | 1.85519E+13 |
|  | 2014 | 1958788233 | 6.90217E+11 | 53327179708 | 1.846E+13 |
|  | 2015 | 1727074196 | 6.24874E+11 | 51252405508 | 1.61321E+13 |
|  | 2016 | 1823650063 | 6.44932E+11 | 48946091849 | 1.56794E+13 |
|  | 2017 | 2026172479 | 6.98131E+11 | 52930978543 | 1.7271E+13 |
|  | 2018 | 2230041645 | 7.38195E+11 | 58249024481 | 1.89541E+13 |
|  | 2019 | 2399081902 | 7.05671E+11 | 60141078135 | 1.836E+13 |
|  | 2020 | 2452168796 | 6.41283E+11 | 64964866446 | 1.71299E+13 |
|  | 2021 | 2822995778 | 7.57066E+11 | 82970404994 | 2.15086E+13 |
| HS854140 | 2001 | 1984989624 | 4.03344E+11 | 6713998933 | 6.06563E+12 |
|  | 2002 | 2436429653 | 4.16729E+11 | 7179332265 | 6.38013E+12 |
|  | 2003 | 3548306844 | 4.72007E+11 | 9932685509 | 7.45788E+12 |
|  | 2004 | 4628904411 | 5.65761E+11 | 13005465288 | 9.02332E+12 |
|  | 2005 | 4796150751 | 5.94941E+11 | 15762738743 | 1.01628E+13 |
|  | 2006 | 5198761465 | 6.46725E+11 | 20416084673 | 1.18798E+13 |
|  | 2007 | 5472218225 | 7.14327E+11 | 27328567002 | 1.36111E+13 |
|  | 2008 | 6189832071 | 7.81412E+11 | 44075301249 | 1.56856E+13 |
|  | 2009 | 4673368026 | 5.80719E+11 | 39324527570 | 1.22547E+13 |
|  | 2010 | 6446197098 | 7.69774E+11 | 72406848962 | 1.49017E+13 |
|  | 2011 | 6604123379 | 8.23184E+11 | 74168174379 | 1.78992E+13 |
|  | 2012 | 5832185723 | 7.9862E+11 | 53816544857 | 1.7838E+13 |
|  | 2013 | 4725686962 | 7.15097E+11 | 50295528204 | 1.85519E+13 |
|  | 2014 | 4535930149 | 6.90217E+11 | 54280798128 | 1.846E+13 |
|  | 2015 | 4032256578 | 6.24874E+11 | 56868728529 | 1.61321E+13 |
|  | 2016 | 3908783367 | 6.44932E+11 | 53121579803 | 1.56794E+13 |
|  | 2017 | 3882803131 | 6.98131E+11 | 52130102560 | 1.7271E+13 |
|  | 2018 | 3956363590 | 7.38195E+11 | 58249024481 | 1.89541E+13 |
|  | 2019 | 3531076700 | 7.05671E+11 | 60141078135 | 1.836E+13 |
|  | 2020 | 3443557632 | 6.41283E+11 | 64964866446 | 1.71299E+13 |
|  | 2021 | 3820645399 | 7.57066E+11 | 82970404994 | 2.15086E+13 |
| HS841990 | 2001 | 127879536 | 4.03344E+11 | 2443086166 | 6.06563E+12 |
|  | 2002 | 128123631 | 4.16729E+11 | 2412170325 | 6.38013E+12 |
|  | 2003 | 151692135 | 4.72007E+11 | 2825555174 | 7.45788E+12 |
|  | 2004 | 244745678 | 5.65761E+11 | 3455050913 | 9.02332E+12 |
|  | 2005 | 334133115 | 5.95E+11 | 4149219879 | 1.02E+13 |
|  | 2006 | 292981574 | 6.47E+11 | 4963291717 | 1.19E+13 |
|  | 2007 | 157481656 | 7.14E+11 | 6841039110 | 1.36E+13 |
|  | 2008 | 249285237 | 7.81E+11 | 7067302136 | 1.57E+13 |
|  | 2009 | 323573893 | 5.81E+11 | 5609714551 | 1.23E+13 |
|  | 2010 | 198335679 | 7.70E+11 | 5523756597 | 1.49E+13 |
|  | 2011 | 236367467 | 8.23E+11 | 6414565689 | 1.79E+13 |
|  | 2012 | 227947739 | 7.99E+11 | 6720957298 | 1.78E+13 |
|  | 2013 | 231451925 | 7.15E+11 | 6816610839 | 1.86E+13 |
|  | 2014 | 176728295 | 6.90E+11 | 6759789698 | 1.85E+13 |
|  | 2015 | 152565224 | 6.25E+11 | 6282411116 | 1.61E+13 |
|  | 2016 | 176525526 | 6.45E+11 | 6147822578 | 1.57E+13 |
|  | 2017 | 187982537 | 6.98E+11 | 6138200431 | 1.73E+13 |
|  | 2018 | 3956363590 | 7.38E+11 | 58249024481 | 1.89541E+13 |
|  | 2019 | 3531076700 | 7.06E+11 | 60141078135 | 1.836E+13 |
|  | 2020 | 3443557632 | 6.41E+11 | 64964866446 | 1.71299E+13 |
|  | 2021 | 3820645399 | 7.57E+11 | 82970404994 | 2.15086E+13 |

Trade data for Singapore

| Code | Year | Total exports of the product from the country | Total exports of the country | Total world exports of the product | Total world export |
| --- | --- | --- | --- | --- | --- |
| HS850440 | 2001 | 346114010 | 1.21754E+11 | 15277808966 | 6.06563E+12 |
|  | 2002 | 319576368 | 1.25177E+11 | 14331199364 | 6.38013E+12 |
|  | 2003 | 380017223 | 1.59963E+11 | 16470599661 | 7.45788E+12 |
|  | 2004 | 462201140 | 1.98633E+11 | 19943301119 | 9.02332E+12 |
|  | 2005 | 441258366 | 2.30344E+11 | 21905426253 | 1.01628E+13 |
|  | 2006 | 518275831 | 2.73382E+11 | 26743646603 | 1.18798E+13 |
|  | 2007 | 677567996 | 3.01595E+11 | 33152966500 | 1.36111E+13 |
|  | 2008 | 1019417222 | 3.41079E+11 | 37779806328 | 1.56856E+13 |
|  | 2009 | 704059904 | 2.7108E+11 | 32380692622 | 1.22547E+13 |
|  | 2010 | 763286356 | 3.5324E+11 | 44068548896 | 1.49017E+13 |
|  | 2011 | 804041173 | 4.16289E+11 | 49441655896 | 1.78992E+13 |
|  | 2012 | 712137101 | 4.15615E+11 | 49668324792 | 1.7838E+13 |
|  | 2013 | 698510076 | 4.19932E+11 | 52352958739 | 1.85519E+13 |
|  | 2014 | 682372124 | 4.15418E+11 | 53327179708 | 1.846E+13 |
|  | 2015 | 770264958 | 3.57941E+11 | 51252405508 | 1.61321E+13 |
|  | 2016 | 800803373 | 3.38082E+11 | 48946091849 | 1.56794E+13 |
|  | 2017 | 770471211 | 3.73255E+11 | 52930978543 | 1.7271E+13 |
|  | 2018 | 923679976 | 4.11743E+11 | 58249024481 | 1.89541E+13 |
|  | 2019 | 858335733 | 3.90332E+11 | 60141078135 | 1.836E+13 |
|  | 2020 | 879142406 | 3.73684E+11 | 64964866446 | 1.71299E+13 |
|  | 2021 | 987769229 | 4.57081E+11 | 82970404994 | 2.15086E+13 |
| HS854140 | 2001 | 194892429 | 1.21754E+11 | 6713998933 | 6.06563E+12 |
|  | 2002 | 240374387 | 1.25177E+11 | 7179332265 | 6.38013E+12 |
|  | 2003 | 314375624 | 1.59963E+11 | 9932685509 | 7.45788E+12 |
|  | 2004 | 328702739 | 1.98633E+11 | 13005465288 | 9.02332E+12 |
|  | 2005 | 317147497 | 2.30344E+11 | 15762738743 | 1.01628E+13 |
|  | 2006 | 444582639 | 2.73382E+11 | 20416084673 | 1.18798E+13 |
|  | 2007 | 500328056 | 3.01595E+11 | 27328567002 | 1.36111E+13 |
|  | 2008 | 737246585 | 3.41079E+11 | 44075301249 | 1.56856E+13 |
|  | 2009 | 673660312 | 2.7108E+11 | 39324527570 | 1.22547E+13 |
|  | 2010 | 1253434117 | 3.5324E+11 | 72406848962 | 1.49017E+13 |
|  | 2011 | 2080715454 | 4.16289E+11 | 74168174379 | 1.78992E+13 |
|  | 2012 | 1585091208 | 4.15615E+11 | 53816544857 | 1.7838E+13 |
|  | 2013 | 1501935234 | 4.19932E+11 | 50295528204 | 1.85519E+13 |
|  | 2014 | 1793650375 | 4.15418E+11 | 54280798128 | 1.846E+13 |
|  | 2015 | 2360762494 | 3.57941E+11 | 56868728529 | 1.61321E+13 |
|  | 2016 | 2605416036 | 3.38082E+11 | 53121579803 | 1.56794E+13 |
|  | 2017 | 2338953630 | 3.73255E+11 | 52130102560 | 1.7271E+13 |
|  | 2018 | 1695184457 | 4.11743E+11 | 58249024481 | 1.89541E+13 |
|  | 2019 | 1542785265 | 3.90332E+11 | 60141078135 | 1.836E+13 |
|  | 2020 | 1810129126 | 3.73684E+11 | 64964866446 | 1.71299E+13 |
|  | 2021 | 2164971755 | 4.57081E+11 | 82970404994 | 2.15086E+13 |
| HS841990 | 2001 | 4554200 | 1.21754E+11 | 2443086166 | 6.06563E+12 |
|  | 2002 | 9447043 | 1.25177E+11 | 2412170325 | 6.38013E+12 |
|  | 2003 | 11480250 | 1.59963E+11 | 2825555174 | 7.45788E+12 |
|  | 2004 | 22969547 | 1.98633E+11 | 3455050913 | 9.02332E+12 |
|  | 2005 | 19424491 | 2.30344E+11 | 4149219879 | 1.01628E+13 |
|  | 2006 | 59982155 | 2.73382E+11 | 4963291717 | 1.18798E+13 |
|  | 2007 | 74021685 | 3.01595E+11 | 6841039110 | 1.36111E+13 |
|  | 2008 | 38153943 | 3.41079E+11 | 7067302136 | 1.56856E+13 |
|  | 2009 | 67242233 | 2.7108E+11 | 5609714551 | 1.22547E+13 |
|  | 2010 | 60230965 | 3.5324E+11 | 5523756597 | 1.49017E+13 |
|  | 2011 | 102088509 | 4.16289E+11 | 6414565689 | 1.78992E+13 |
|  | 2012 | 132661484 | 4.15615E+11 | 6720957298 | 1.7838E+13 |
|  | 2013 | 108327716 | 4.19932E+11 | 6816610839 | 1.85519E+13 |
|  | 2014 | 126733224 | 4.15418E+11 | 6759789698 | 1.846E+13 |
|  | 2015 | 131168760 | 3.57941E+11 | 6282411116 | 1.61321E+13 |
|  | 2016 | 73785578 | 3.38082E+11 | 6147822578 | 1.56794E+13 |
|  | 2017 | 78243268 | 3.73255E+11 | 6138200431 | 1.7271E+13 |
|  | 2018 | 101870245 | 4.11743E+11 | 6367541909 | 1.89541E+13 |
|  | 2019 | 84298709 | 3.90332E+11 | 6388526721 | 1.836E+13 |
|  | 2020 | 192866230 | 3.73684E+11 | 5938406474 | 1.71299E+13 |
|  | 2021 | 139430632 | 4.57081E+11 | 6724014953 | 2.15086E+13 |

Chile's trade data

| Code | Year | Total exports of the product from the country | Total exports of the country | Total world exports of the product | Total world export |
| --- | --- | --- | --- | --- | --- |
| HS850440 | 2001 | 524887 | 18745414508 | 15277808966 | 6.06563E+12 |
|  | 2002 | 737200 | 17423088183 | 14331199364 | 6.38013E+12 |
|  | 2003 | 1349476 | 21650905646 | 16470599661 | 7.45788E+12 |
|  | 2004 | 2015720 | 33025406983 | 19943301119 | 9.02332E+12 |
|  | 2005 | 2790767 | 41972988436 | 21905426253 | 1.01628E+13 |
|  | 2006 | 3681319 | 60596327585 | 26743646603 | 1.18798E+13 |
|  | 2007 | 2435069 | 68560429272 | 33152966500 | 1.36111E+13 |
|  | 2008 | 3064060 | 64507601394 | 37779806328 | 1.56856E+13 |
|  | 2009 | 3655886 | 55458960013 | 32380692622 | 1.22547E+13 |
|  | 2010 | 4701194 | 71106105854 | 44068548896 | 1.49017E+13 |
|  | 2011 | 7706740 | 81437589325 | 49441655896 | 1.78992E+13 |
|  | 2012 | 7333353 | 78062994755 | 49668324792 | 1.7838E+13 |
|  | 2013 | 6662644 | 76769729414 | 52352958739 | 1.85519E+13 |
|  | 2014 | 5031525 | 75083496948 | 53327179708 | 1.846E+13 |
|  | 2015 | 4972893 | 62033060412 | 51252405508 | 1.61321E+13 |
|  | 2016 | 3408237 | 60717528242 | 48946091849 | 1.56794E+13 |
|  | 2017 | 14007101 | 68858401595 | 52930978543 | 1.7271E+13 |
|  | 2018 | 6092929 | 75481713821 | 58249024481 | 1.89541E+13 |
|  | 2019 | 4089004 | 69681396828 | 60141078135 | 1.836E+13 |
|  | 2020 | 3877435 | 73479930615 | 64964866446 | 1.71299E+13 |
|  | 2021 | 6088736 | 94705083260 | 82970404994 | 2.15086E+13 |
| HS854140 | 2001 | 22923 | 18745414508 | 6713998933 | 6.06563E+12 |
|  | 2002 | 5591 | 17423088183 | 7179332265 | 6.38013E+12 |
|  | 2003 | 19823 | 21650905646 | 9932685509 | 7.45788E+12 |
|  | 2004 | 54087 | 33025406983 | 13005465288 | 9.02332E+12 |
|  | 2005 | 30758 | 41972988436 | 15762738743 | 1.01628E+13 |
|  | 2006 | 30430 | 60596327585 | 20416084673 | 1.18798E+13 |
|  | 2007 | 35566 | 68560429272 | 27328567002 | 1.36111E+13 |
|  | 2008 | 91522 | 64507601394 | 44075301249 | 1.56856E+13 |
|  | 2009 | 119557 | 55458960013 | 39324527570 | 1.22547E+13 |
|  | 2010 | 56821 | 71106105854 | 72406848962 | 1.49017E+13 |
|  | 2011 | 106563 | 81437589325 | 74168174379 | 1.78992E+13 |
|  | 2012 | 158907 | 78062994755 | 53816544857 | 1.7838E+13 |
|  | 2013 | 222176 | 76769729414 | 50295528204 | 1.85519E+13 |
|  | 2014 | 170937 | 75083496948 | 54280798128 | 1.846E+13 |
|  | 2015 | 80467 | 62033060412 | 56868728529 | 1.61321E+13 |
|  | 2016 | 14461512 | 60717528242 | 53121579803 | 1.56794E+13 |
|  | 2017 | 1710303 | 68858401595 | 52130102560 | 1.7271E+13 |
|  | 2018 | 2831320 | 7.55E+10 | 58249024481 | 1.90E+13 |
|  | 2019 | 282618 | 6.97E+10 | 60141078135 | 1.84E+13 |
|  | 2020 | 179801 | 7.35E+10 | 64964866446 | 1.71E+13 |
|  | 2021 | 277103 | 9.47E+10 | 82970404994 | 2.15E+13 |
| HS841990 | 2001 | 514473 | 1.87E+10 | 2443086166 | 6.07E+12 |
|  | 2002 | 237379 | 1.74E+10 | 2412170325 | 6.38E+12 |
|  | 2003 | 282101 | 2.17E+10 | 2825555174 | 7.46E+12 |
|  | 2004 | 502435 | 3.30E+10 | 3455050913 | 9.02E+12 |
|  | 2005 | 1551618 | 4.20E+10 | 4149219879 | 1.02E+13 |
|  | 2006 | 1128939 | 6.06E+10 | 4963291717 | 1.19E+13 |
|  | 2007 | 716054 | 6.86E+10 | 6841039110 | 1.36E+13 |
|  | 2008 | 2079315 | 6.45E+10 | 7067302136 | 1.57E+13 |
|  | 2009 | 1874204 | 5.55E+10 | 5609714551 | 1.23E+13 |
|  | 2010 | 3390698 | 7.11E+10 | 5523756597 | 1.49E+13 |
|  | 2011 | 646891 | 8.14E+10 | 6414565689 | 1.79E+13 |
|  | 2012 | 1016281 | 7.81E+10 | 6720957298 | 1.78E+13 |
|  | 2013 | 1350273 | 7.68E+10 | 6816610839 | 1.86E+13 |
|  | 2014 | 223973 | 7.51E+10 | 6759789698 | 1.85E+13 |
|  | 2015 | 1013887 | 6.20E+10 | 6282411116 | 1.61E+13 |
|  | 2016 | 646573 | 6.07E+10 | 6147822578 | 1.57E+13 |
|  | 2017 | 531625 | 6.89E+10 | 6138200431 | 1.73E+13 |
|  | 2018 | 765437 | 75481713821 | 58249024481 | 1.89541E+13 |
|  | 2019 | 712012 | 69681396828 | 60141078135 | 1.836E+13 |
|  | 2020 | 1759904 | 73479930615 | 64964866446 | 1.71299E+13 |
|  | 2021 | 1432088 | 94705083260 | 82970404994 | 2.15086E+13 |

Vietnam's trade data

| Code | Year | Total exports of the product from the country | Total exports of the country | Total world exports of the product | Total world export |
| --- | --- | --- | --- | --- | --- |
| HS850440 | 2001 | 37348 | 15029192447 | 15277808966 | 6.06563E+12 |
|  | 2002 | 826496 | 16706052543 | 14331199364 | 6.38013E+12 |
|  | 2003 | 435295 | 20149323745 | 16470599661 | 7.45788E+12 |
|  | 2004 | 296583 | 26485034706 | 19943301119 | 9.02332E+12 |
|  | 2005 | 880574 | 32447129167 | 21905426253 | 1.01628E+13 |
|  | 2006 | 1969592 | 39826222802 | 26743646603 | 1.18798E+13 |
|  | 2007 | 1944088 | 48561343186 | 33152966500 | 1.36111E+13 |
|  | 2008 | 3516376 | 62685129696 | 37779806328 | 1.56856E+13 |
|  | 2009 | 2776530 | 57096274457 | 32380692622 | 1.22547E+13 |
|  | 2010 | 9021571 | 72236665000 | 44068548896 | 1.49017E+13 |
|  | 2011 | 20434233 | 96905673959 | 49441655896 | 1.78992E+13 |
|  | 2012 | 32005894 | 1.14529E+11 | 49668324792 | 1.7838E+13 |
|  | 2013 | 95514819 | 1.32033E+11 | 52352958739 | 1.85519E+13 |
|  | 2014 | 149927265 | 1.50217E+11 | 53327179708 | 1.846E+13 |
|  | 2015 | 356883998 | 1.62017E+11 | 51252405508 | 1.61321E+13 |
|  | 2016 | 511638821 | 1.76581E+11 | 48946091849 | 1.56794E+13 |
|  | 2017 | 537758902 | 2.15119E+11 | 52930978543 | 1.7271E+13 |
|  | 2018 | 566388614 | 2.43699E+11 | 58249024481 | 1.89541E+13 |
|  | 2019 | 820350507 | 2.6461E+11 | 60141078135 | 1.836E+13 |
|  | 2020 | 1185593070 | 2.81441E+11 | 64964866446 | 1.71299E+13 |
|  | 2021 | 3248201334 | 3.35793E+11 | 82970404994 | 2.15086E+13 |
| HS854140 | 2001 | 462 | 15029192447 | 6713998933 | 6.06563E+12 |
|  | 2002 | 302 | 16706052543 | 7179332265 | 6.38013E+12 |
|  | 2003 | 3409 | 20149323745 | 9932685509 | 7.45788E+12 |
|  | 2004 | 2076 | 26485034706 | 13005465288 | 9.02332E+12 |
|  | 2005 | 2774874 | 32447129167 | 15762738743 | 1.01628E+13 |
|  | 2006 | 5394101 | 39826222802 | 20416084673 | 1.18798E+13 |
|  | 2007 | 6794485 | 48561343186 | 27328567002 | 1.36111E+13 |
|  | 2008 | 7863182 | 62685129696 | 44075301249 | 1.56856E+13 |
|  | 2009 | 1958377 | 57096274457 | 39324527570 | 1.22547E+13 |
|  | 2010 | 12884803 | 72236665000 | 72406848962 | 1.49017E+13 |
|  | 2011 | 20698613 | 96905673959 | 74168174379 | 1.78992E+13 |
|  | 2012 | 111705778 | 1.14529E+11 | 53816544857 | 1.7838E+13 |
|  | 2013 | 135238612 | 1.32033E+11 | 50295528204 | 1.85519E+13 |
|  | 2014 | 92150444 | 1.50217E+11 | 54280798128 | 1.846E+13 |
|  | 2015 | 551223437 | 1.62017E+11 | 56868728529 | 1.61321E+13 |
|  | 2016 | 1617755858 | 1.76581E+11 | 53121579803 | 1.56794E+13 |
|  | 2017 | 2325298765 | 2.15119E+11 | 52130102560 | 1.7271E+13 |
|  | 2018 | 2000068487 | 2.44E+11 | 58249024481 | 1.90E+13 |
|  | 2019 | 3507961660 | 2.65E+11 | 60141078135 | 1.84E+13 |
|  | 2020 | 4460420504 | 2.81E+11 | 64964866446 | 1.71E+13 |
|  | 2021 | 4789911918 | 3.36E+11 | 82970404994 | 2.15E+13 |
| HS841990 | 2001 | 57107 | 1.50E+10 | 2443086166 | 6.07E+12 |
|  | 2002 | 121539 | 1.67E+10 | 2412170325 | 6.38E+12 |
|  | 2003 | 229225 | 2.01E+10 | 2825555174 | 7.46E+12 |
|  | 2004 | 35402 | 2.65E+10 | 3455050913 | 9.02E+12 |
|  | 2005 | 433167 | 3.24E+10 | 4149219879 | 1.02E+13 |
|  | 2006 | 4843099 | 3.98E+10 | 4963291717 | 1.19E+13 |
|  | 2007 | 10382821 | 4.86E+10 | 6841039110 | 1.36E+13 |
|  | 2008 | 6871399 | 6.27E+10 | 7067302136 | 1.57E+13 |
|  | 2009 | 2628502 | 5.71E+10 | 5609714551 | 1.23E+13 |
|  | 2010 | 5387634 | 7.22E+10 | 5523756597 | 1.49E+13 |
|  | 2011 | 2850830 | 9.69E+10 | 6414565689 | 1.79E+13 |
|  | 2012 | 100714818 | 1.15E+11 | 6720957298 | 1.78E+13 |
|  | 2013 | 10661425 | 1.32E+11 | 6816610839 | 1.86E+13 |
|  | 2014 | 4273685 | 1.50E+11 | 6759789698 | 1.85E+13 |
|  | 2015 | 7051792 | 1.62E+11 | 6282411116 | 1.61E+13 |
|  | 2016 | 11439727 | 1.77E+11 | 6147822578 | 1.57E+13 |
|  | 2017 | 22603315 | 2.15E+11 | 6138200431 | 1.73E+13 |
|  | 2018 | 30597617 | 2.43699E+11 | 6367541909 | 1.89541E+13 |
|  | 2019 | 20537931 | 2.6461E+11 | 6388526721 | 1.836E+13 |
|  | 2020 | 15695410 | 2.81441E+11 | 5938406474 | 1.71299E+13 |
|  | 2021 | 21032428 | 3.35793E+11 | 6724014953 | 2.15086E+13 |

World imports of solar photovoltaic products

| Year | HS841990 | HS850440 | HS854140 |
| --- | --- | --- | --- |
| 2000 | 2431759350 | 18079541523 | 7260436976 |
| 2001 | 2311623326 | 16713612910 | 6919940853 |
| 2002 | 2241073597 | 16472925788 | 7198300532 |
| 2003 | 2678791028 | 18960589407 | 9368251334 |
| 2004 | 3171053188 | 22563962193 | 12687842890 |
| 2005 | 3761226398 | 25452765060 | 15843834335 |
| 2006 | 4425609951 | 29854662936 | 19862715907 |
| 2007 | 5714821423 | 36204332113 | 26167028433 |
| 2008 | 6539384506 | 40407613821 | 42598100689 |
| 2009 | 5039369454 | 33925529483 | 38594213156 |
| 2010 | 5179330248 | 71329194489 | 71329194489 |
| 2011 | 5659392706 | 73460950934 | 73460950934 |
| 2012 | 5971304461 | 54424116318 | 54424116318 |
| 2013 | 6388532426 | 51218727871 | 51218727871 |
| 2014 | 6477150984 | 54638011069 | 54638011069 |
| 2015 | 5958501969 | 51131638686 | 55717795707 |
| 2016 | 5708107625 | 50335874682 | 54959972819 |
| 2017 | 6268920325 | 54438460658 | 54708875474 |
| 2018 | 6866614489 | 59337029730 | 53181681090 |
| 2019 | 6502331525 | 61200863136 | 57088683587 |
| 2020 | 6202974914 | 64746167496 | 58658554527 |
| 2021 | 6662551899 | 78757498212 | 72449840682 |

Various metrics used to obtain weights

| Year | GDP (USD) | Population | Solar power generation (gigawatt-hours) | The proportion of renewable energy consumption in terminal energy consumption | Total renewable energy generation (GWH) |
| --- | --- | --- | --- | --- | --- |
| 2001 | 4374711762944.00 | 127149000 | 1003 | 0.0361 | 95000 |
| 2002 | 418284547904 | 127445000 | 1341 | 0.0376 | 95001 |
| 2003 | 4519561789440 | 127718000 | 1243 | 0.0412 | 114000 |
| 2004 | 4893115940864.00 | 127761000 | 1001 | 0.0421 | 115000 |
| 2005 | 4831467012096.00 | 127773000 | 1597 | 0.04 | 99000 |
| 2006 | 4601663193088 | 127854000 | 1678 | 0.0435 | 110854 |
| 2007 | 4579751100416.00 | 128001000 | 1690 | 0.0422 | 985345 |
| 2008 | 5106678890496.00 | 128063000 | 2063 | 0.0434 | 1000000 |
| 2009 | 5289492873216.00 | 128047000 | 2154 | 0.045 | 105000 |
| 2010 | 5759071944704.00 | 128070000 | 2265 | 0.0455 | 110660 |
| 2011 | 6233147310080.00 | 127833000 | 3144 | 0.0464 | 110661 |
| 2012 | 6272363003904.00 | 127629000 | 6524 | 0.047 | 103000 |
| 2013 | 5212328165376.00 | 127445000 | 15788 | 0.0491 | 118654 |
| 2014 | 4896994623488.00 | 127276000 | 21757 | 0.0532 | 130089 |
| 2015 | 4444930441216.00 | 127141000 | 35684 | 0.062 | 146853 |
| 2016 | 5003677794304.00 | 126995000 | 43685 | 0.0632 | 150709 |
| 2017 | 4930837413888.00 | 126786000 | 54963 | 0.0655 | 165908 |
| 2018 | 4830837413889 | 126634232 | 61343 | 0.0755 | 175869 |
| 2019 | 5030837413890 | 126752353 | 64634 | 0.0785 | 190125 |
| 2020 | 5134792374931 | 125231786 | 66353 | 0.0855 | 201321 |
| 2021 | 5230832444511 | 125124234 | 69645 | 0.0913 | 218424 |
| 2001 | 32685199360.00 | 80742500 | 1 | 0.56 | 24000 |
| 2002 | 35064107008.00 | 81534400 | 3 | 0.515 | 24170 |
| 2003 | 39552512000.00 | 82301600 | 4 | 0.504 | 24268 |
| 2004 | 45427855360.00 | 83062800 | 3 | 0.46 | 24178 |
| 2005 | 57633255424.00 | 83832700 | 5 | 0.444 | 24668 |
| 2006 | 66371665920.00 | 84617500 | 6 | 0.45 | 24746 |
| 2007 | 77414424576.00 | 85419600 | 6 | 0.42 | 30975 |
| 2008 | 99130302464.00 | 86243400 | 5 | 0.4 | 32789 |
| 2009 | 106014662656.00 | 87092200 | 5.5 | 0.38 | 31647 |
| 2010 | 115931750400.00 | 87967700 | 5 | 0.35 | 41354 |
| 2011 | 135539441664.00 | 88871400 | 6 | 0.36 | 47579 |
| 2012 | 155819999232.00 | 89801900 | 8 | 0.377 | 57546 |
| 2013 | 171222024192.00 | 90752600 | 7 | 0.37 | 58642 |
| 2014 | 186204651520.00 | 91713800 | 9 | 0.365 | 59746 |
| 2015 | 193241104384.00 | 92677100 | 7 | 0.3 | 55690 |
| 2016 | 205276168192.00 | 93640400 | 9 | 0.22 | 61356 |
| 2017 | 223779864576.00 | 94600600 | 10 | 0.25 | 92467 |
| 2018 | 243231743183 | 95021311 | 11 | 0.23 | 102132 |
| 2019 | 253823791378 | 96301123 | 13 | 0.24 | 109213 |
| 2020 | 263132132323 | 97472321 | 14 | 0.25 | 110321 |
| 2021 | 273195785632 | 98313123 | 15 | 0.22 | 114232 |
| 2001 | 32685199360.00 | 80742500 | 1 | 0.56 | 24000 |
| 2002 | 35064107008.00 | 81534400 | 3 | 0.515 | 24170 |
| 2003 | 39552512000.00 | 82301600 | 4 | 0.504 | 24268 |
| 2004 | 45427855360.00 | 83062800 | 3 | 0.46 | 24178 |
| 2005 | 57633255424.00 | 83832700 | 5 | 0.444 | 24668 |
| 2001 | 32685199360.00 | 15516100 | 2 | 0.32 | 23535 |
| 2002 | 35064107008.00 | 15684400 | 3 | 0.335 | 24567 |
| 2003 | 39552512000.00 | 15849600 | 4 | 0.318 | 24153 |
| 2004 | 45427855360.00 | 16015000 | 5 | 0.325 | 24763 |
| 2005 | 57633255424.00 | 16182700 | 4 | 0.33 | 28467 |
| 2006 | 66371665920.00 | 16354500 | 7 | 0.305 | 30146 |
| 2007 | 77414424576.00 | 16530200 | 4 | 0.31 | 25367 |
| 2008 | 99130302464.00 | 16708300 | 9 | 0.32 | 27476 |
| 2009 | 106014662656.00 | 16886200 | 10 | 0.32 | 29646 |
| 2010 | 115931750400.00 | 17062500 | 11 | 0.27 | 24665 |
| 2011 | 135539441664.00 | 17233600 | 12 | 0.285 | 25942 |
| 2012 | 155819999232.00 | 17400400 | 87 | 0.302 | 25134 |
| 2013 | 171222024192.00 | 17571500 | 100 | 0.3 | 25854 |
| 2014 | 186204651520.00 | 17759000 | 450 | 0.27 | 30537 |
| 2015 | 193241104384.00 | 17969400 | 1250 | 0.25 | 32745 |
| 2016 | 205276168192.00 | 18209100 | 2500 | 0.245 | 34157 |
| 2017 | 223779864576 | 18470400 | 4170 | 0.285 | 35000 |
| 2018 | 232266954443 | 18632321 | 5013 | 0.301 | 35132 |
| 2019 | 242323245452 | 18912321 | 5132 | 0.35 | 36231 |
| 2020 | 251234796431 | 19023131 | 5931 | 0.391 | 36932 |
| 2021 | 268542456987 | 19523186 | 6043 | 0.431 | 37231 |
| 2001 | 70979919872.00 | 15516100 | 2 | 0.32 | 23535 |
| 2002 | 69736808448.00 | 15684400 | 3 | 0.335 | 24567 |
| 2003 | 75643461632.00 | 15849600 | 4 | 0.318 | 24153 |
| 2004 | 99210395648.00 | 16015000 | 5 | 0.325 | 24763 |
| 2005 | 122964811776.00 | 16182700 | 4 | 0.33 | 28467 |
| 2006 | 154788020224.00 | 16354500 | 7 | 0.305 | 30146 |
| 2007 | 173605961728.00 | 16530200 | 4 | 0.31 | 25367 |
| 2008 | 179638501376.00 | 16708300 | 9 | 0.32 | 27476 |
| 2009 | 172389498880.00 | 16886200 | 10 | 0.32 | 29646 |
| 2010 | 218537558016.00 | 17062500 | 11 | 0.27 | 24665 |
| 2011 | 252251996160.00 | 17233600 | 12 | 0.285 | 25942 |
| 2012 | 267122327552.00 | 17400400 | 87 | 0.302 | 25134 |
| 2013 | 278384345088.00 | 17571500 | 100 | 0.3 | 25854 |
| 2014 | 260541644800.00 | 17759000 | 450 | 0.27 | 30537 |
| 2015 | 243919077376.00 | 17969400 | 1250 | 0.25 | 32745 |
| 2016 | 250440155136.00 | 18209100 | 2500 | 0.245 | 34157 |
| 2017 | 277034663936.00 | 18470400 | 4170 | 0.285 | 35000 |
| 2018 | 283158374323 | 18692323 | 5013 | 0.301 | 35132 |
| 2019 | 291324232433 | 18942313 | 5132 | 0.35 | 36231 |
| 2020 | 303143543573 | 19323241 | 5931 | 0.391 | 36932 |
| 2021 | 318432847328 | 2013123 | 6043 | 0.431 | 37231 |
| 2001 | 70979919872.00 | 4138010 | 0 | 0.006 | 1413 |
| 2002 | 69736808448.00 | 4175950 | 0 | 0.0058 | 1414 |
| 2003 | 75643461632.00 | 4114830 | 0 | 0.0059 | 1415 |
| 2004 | 99210395648.00 | 4166660 | 0 | 0.0055 | 1416 |
| 2005 | 122964811776.00 | 4265760 | 0 | 0.0056 | 1417 |
| 2006 | 154788020224.00 | 4401360 | 0 | 0.0054 | 1418 |
| 2007 | 173605961728.00 | 4588600 | 0 | 0.005 | 1419 |
| 2008 | 179638501376.00 | 4839400 | 2 | 0.0056 | 1420 |
| 2009 | 172389498880.00 | 4987570 | 4 | 0.0048 | 1154 |
| 2010 | 218537558016.00 | 5076730 | 7 | 0.0047 | 1523 |
| 2011 | 252251996160.00 | 5183690 | 8 | 0.0048 | 1557 |
| 2012 | 267122327552.00 | 5312440 | 9 | 0.0054 | 1575 |
| 2013 | 278384345088.00 | 5399160 | 15 | 0.0058 | 1785 |
| 2014 | 260541644800.00 | 5469720 | 23 | 0.006 | 2013 |
| 2015 | 243919077376.00 | 5535000 | 53 | 0.0063 | 2143 |
| 2016 | 250440155136.00 | 5607280 | 102 | 0.0064 | 2462 |
| 2017 | 277034663936.00 | 5612250 | 121 | 0.0064 | 2313 |
| 2018 | 283158374323 | 5693231 | 137 | 0.0071 | 2454 |
| 2019 | 291324232433 | 6031321 | 156 | 0.0079 | 2397 |
| 2020 | 303143543573 | 6231831 | 189 | 0.0081 | 2531 |
| 2021 | 318432847328 | 6312131 | 225 | 0.0092 | 2631 |
| 2001 | 89794945024.00 | 100298000 | 1 | 0.112 | 37432 |
| 2002 | 92537749504.00 | 101685000 | 12 | 0.108 | 32142 |
| 2003 | 97645445120.00 | 103081000 | 13 | 0.101 | 29233 |
| 2004 | 115035496448.00 | 104515000 | 14 | 0.1015 | 35634 |
| 2005 | 127807619072.00 | 106005000 | 15 | 0.102 | 39253 |
| 2006 | 148630372352.00 | 107560000 | 16 | 0.099 | 39956 |
| 2007 | 180941946880.00 | 109171000 | 29 | 0.095 | 38135 |
| 2008 | 193611988992.00 | 110815000 | 30 | 0.098 | 48947 |
| 2009 | 194152284160.00 | 112464000 | 31 | 0.092 | 34253 |
| 2010 | 239809380352.00 | 114093000 | 32 | 0.0937 | 45634 |
| 2011 | 279351164928.00 | 115695000 | 33 | 0.0911 | 43535 |
| 2012 | 295087210496.00 | 117274000 | 25 | 0.09 | 42135 |
| 2013 | 307576373248.00 | 118827000 | 100 | 0.092 | 40242 |
| 2014 | 314851164160.00 | 120355000 | 122 | 0.098 | 51245 |
| 2015 | 308004159488.00 | 121858000 | 213 | 0.092 | 48253 |
| 2016 | 318763794432.00 | 123333000 | 253 | 0.0926 | 49134 |
| 2017 | 343337762816.00 | 124777000 | 1212 | 0.1 | 51253 |
| 2018 | 358769458355 | 134231331 | 1942 | 0.14 | 53132 |
| 2019 | 360684028943 | 147392345 | 1842 | 0.31 | 54231 |
| 2020 | 363859453453 | 164242343 | 2043 | 0.42 | 56323 |
| 2021 | 384243414567 | 174232131 | 2313 | 0.34 | 59313 |
| 2001 | 756706312192.00 | 100298000 | 1 | 0.112 | 37432 |
| 2002 | 772106354688.00 | 101685000 | 12 | 0.108 | 32142 |
| 2003 | 729336315904.00 | 103081000 | 13 | 0.101 | 29233 |
| 2004 | 782240579584.00 | 104515000 | 14 | 0.1015 | 35634 |
| 2005 | 877476249600.00 | 106005000 | 15 | 0.102 | 39253 |
| 2006 | 975387099136.00 | 107560000 | 16 | 0.099 | 39956 |
| 2007 | 1052696313856.00 | 109171000 | 29 | 0.095 | 38135 |
| 2008 | 1109988999168.00 | 110815000 | 30 | 0.098 | 48947 |
| 2009 | 900045340672.00 | 112464000 | 31 | 0.092 | 34253 |
| 2010 | 1057801306112.00 | 114093000 | 32 | 0.0937 | 45634 |
| 2011 | 1180489613312.00 | 115695000 | 33 | 0.0911 | 43535 |
| 2012 | 1201089937408.00 | 117274000 | 25 | 0.09 | 42135 |
| 2013 | 1274443071488.00 | 118827000 | 100 | 0.092 | 40242 |
| 2014 | 1315351166976.00 | 120355000 | 122 | 0.098 | 51245 |
| 2015 | 1171867566080.00 | 121858000 | 213 | 0.092 | 48253 |
| 2016 | 1078490628096.00 | 123333000 | 253 | 0.0926 | 49134 |
| 2017 | 1158913064960.00 | 124777000 | 1212 | 0.1 | 51253 |
| 2018 | 119423134349 | 125329831 | 1321 | 0.13 | 53234 |
| 2019 | 129467237249 | 126423115 | 1742 | 0.1423 | 54232 |
| 2020 | 134823y91341 | 129424453 | 1831 | 0.1531 | 56313 |
| 2021 | 139424231245 | 130323134 | 1942 | 0.16 | 57313 |
| 2001 | 92783943680.00 | 23709100 | 13 | 0.0423 | 8142 |
| 2002 | 100845527040.00 | 24208400 | 21 | 0.0365 | 7365 |
| 2003 | 110202372096.00 | 24698800 | 13 | 0.0332 | 7132 |
| 2004 | 124749471744.00 | 25190600 | 5 | 0.0317 | 6953 |
| 2005 | 143534096384.00 | 25690600 | 5 | 0.03 | 7353 |
| 2006 | 162691235840.00 | 26202000 | 7 | 0.031 | 7535 |
| 2007 | 193547829248.00 | 26720400 | 35 | 0.029 | 7524 |
| 2008 | 230813892608.00 | 27236000 | 43 | 0.0302 | 9353 |
| 2009 | 202257629184.00 | 27735000 | 24 | 0.0222 | 8535 |
| 2010 | 255016615936.00 | 28208000 | 21 | 0.02 | 8563 |
| 2011 | 297951952896.00 | 28651000 | 42 | 0.0214 | 9843 |
| 2012 | 314443137024.00 | 29068200 | 24 | 0.027 | 10243 |
| 2013 | 323277160448.00 | 29468900 | 40 | 0.029 | 12434 |
| 2014 | 338061950976.00 | 29866600 | 200 | 0.031 | 15362 |
| 2015 | 301354811392.00 | 30271000 | 285 | 0.033 | 17857 |
| 2016 | 301255393280.00 | 30684700 | 385 | 0.044 | 21524 |
| 2017 | 319112151040.00 | 31104700 | 412 | 0.052 | 28253 |
| 2018 | 328319748173 | 32813671 | 474 | 0.064 | 29313 |
| 2019 | 354382313274 | 33947284 | 498 | 0.074 | 30312 |
| 2020 | 374291492431 | 34826381 | 513 | 0.098 | 31873 |
| 2021 | 394231342311 | 35873914 | 567 | 0.131 | 32133 |
